# Carcinoma-associated fibroblast-derived lysyl oxidase-rich extracellular vesicles mediate collagen crosslinking and promote epithelial-mesenchymal transition via p-FAK/p-paxillin/YAP signaling

**DOI:** 10.1038/s41368-023-00236-1

**Published:** 2023-08-02

**Authors:** Xue Liu, Jiao Li, Xuesong Yang, Xiaojie Li, Jing Kong, Dongyuan Qi, Fuyin Zhang, Bo Sun, Yuehua Liu, Tingjiao Liu

**Affiliations:** 1 Department of Oral Pathology, Shanghai Stomatological Hospital & School of Stomatology, Fudan University, Shanghai, China; 2 Shanghai Key Laboratory of Craniomaxillofacial Development and Diseases, Fudan University, Shanghai, China; 3 Department of Orthodontics, Shanghai Stomatological Hospital & School of Stomatology, Fudan University, Shanghai, China; 4 School of Stomatology, Dalian Medical University, Dalian, China; 5 Department of Biochemistry and Molecular Biology, Liaoning Provincial Core Lab of Glycobiology and Glycoengineering, Dalian Medical University, Dalian, China; 6 Department of Oral Surgery, the First Affiliated Hospital of Dalian Medical University, Dalian, China; 7 Department of Oral Surgery, the Second Affiliated Hospital of Dalian Medical University, Dalian, China

**Keywords:** Cancer microenvironment, Oral cancer

## Abstract

Carcinoma-associated fibroblasts (CAFs) are the main cellular components of the tumor microenvironment and promote cancer progression by modifying the extracellular matrix (ECM). The tumor-associated ECM is characterized by collagen crosslinking catalyzed by lysyl oxidase (LOX). Small extracellular vesicles (sEVs) mediate cell-cell communication. However, the interactions between sEVs and the ECM remain unclear. Here, we demonstrated that sEVs released from oral squamous cell carcinoma (OSCC)-derived CAFs induce collagen crosslinking, thereby promoting epithelial-mesenchymal transition (EMT). CAF sEVs preferably bound to the ECM rather than being taken up by fibroblasts and induced collagen crosslinking, and a LOX inhibitor or blocking antibody suppressed this effect. Active LOX (αLOX), but not the LOX precursor, was enriched in CAF sEVs and interacted with periostin, fibronectin, and bone morphogenetic protein-1 on the surface of sEVs. CAF sEV-associated integrin α2β1 mediated the binding of CAF sEVs to collagen I, and blocking integrin α2β1 inhibited collagen crosslinking by interfering with CAF sEV binding to collagen I. CAF sEV-induced collagen crosslinking promoted the EMT of OSCC through FAK/paxillin/YAP pathway. Taken together, these findings reveal a novel role of CAF sEVs in tumor ECM remodeling, suggesting a critical mechanism for CAF-induced EMT of cancer cells.

## Introduction

Carcinoma-associated fibroblasts (CAFs) are often referred to as activated fibroblasts and they are abundant in the tumor stroma.^1^ CAFs have important functions, including the deposition of extracellular matrix (ECM) constituents and the regulation of tumor cell proliferation and invasion.^2,3^ Collagen, the most abundant ECM component, provides structural integrity and tensile strength to human tissues.^4,5^ The tumor-associated ECM is characterized by collagen crosslinking that provides stability by assembling collagen into thick fibers, thereby increasing the stiffness of the ECM.^6,7^ Increased ECM stiffness is present in various cancers and stimulates epithelial–mesenchymal transition (EMT) of cancer cells.^8–12^ However, the mechanisms by which CAFs drive collagen crosslinking and thus affect tumor cells remain to be fully elucidated.

Lysyl oxidase (LOX) catalyzes the crosslinking of collagen fibrils to form structurally stable collagen I fibers.^13–15^ Collagen initially forms immature crosslinks including dihydroxylysino-norleucine (DHLNL) and hydroxylysino-norleucine (HLNL). These immature forms react with another telopeptide residue to form mature crosslinks such as pyridinoline (PYD).^6,16,17^ LOX is synthesized as a preproenzyme containing an N-terminal signal peptide sequence, a propeptide, and a catalytic domain. The signal peptide is removed intracellularly to generate proLOX, which contains the propeptide and the catalytic domain.^18,19^ The propeptide is required for the exit of proLOX from the endoplasmic reticulum. The proLOX is processed extracellularly by bone morphogenetic protein-1 (BMP-1), resulting in the release of the catalytic domain, a biologically active form of LOX (αLOX).^20,21^ Periostin (POSTN), a secretory protein expressed in collagen rich fibrous tissues, provides a specific microenvironment to regulate LOX catalytic activity.^22,23^ POSTN is composed of three domains: an amino-terminal EMI domain that interacts with fibronectin (FN), a tandem repeat of four FAS1 domains that bind to BMP-1, and a carboxyl-terminal domain.^22,23^ POSTN recruits BMP-1 onto the FN matrix to enhance LOX activity for collagen crosslinking.^24^ Extracellular vesicles (EVs) have attracted considerable attention because of their capacity for carrying proteins, lipids, and nucleic acids and transferring their cargo to recipient cells.^25–27^ EVs are classified into two main categories according to their biogenesis: exosomes and microvesicles.^28^ Exosomes are produced through the fusion of multivesicular bodies with the plasma membrane,^29^ whereas microvesicles are secreted by plasma membrane shedding.^30,31^ In cases in which the intercellular origin of EVs is unknown, size-based EV nomenclature is used as follows: EVs smaller than 200 nm in diameter are defined as small EVs (sEVs), whereas EVs between 200 and 1000 nm in diameter are defined as large EVs.^32^ The role of sEVs in mediating cell-cell communications has been demonstrated; however, whether sEVs mediate the communication between cells and the ECM remains unclear.^33,34^

Excessive cross-linking of collagen I causes ECM stiffening, which changes the mechanical stress microenvironment of surrounding cells.^35^ Yes-associated Protein (YAP) is known as the primary down-stream effector of cellular perception of ECM stiffness, which indirectly leads to the translocation of YAP from the cytoplasm to the nucleus in non-canonical mechanotransduction pathway.^35^ As a universal mechanosensor and mechanoeffector, YAP has been recently reported to mediate EMT in mammary epithelial cells.^36,37^ Therefore, we attempted to speculate that ECM stiffness can modulate EMT of OSCC through YAP-mediated-mechanotransduction.

In this study, we isolated primary CAFs from human oral squamous cell carcinoma (OSCC) specimens to investigate the role of CAF-derived sEVs in ECM remodeling. We demonstrated that αLOX interacts with POSTN and BMP-1 on the surface of CAF sEVs. The sEV-associated αLOX was enzymatically active and induced collagen crosslinking directly. In addition, we verified that CAF sEVs detect collagen I via their surface receptor integrin α2β1. The results indicate that sEV-αLOX promotes collagen crosslinking, thereby inducing EMT of OSCC via p-FAK/p-paxillin/YAP signaling.

## Results

### Clinical values of collagen crosslinking and LOX-rich on the surface of CAF sEVs

**Fig. 1:**
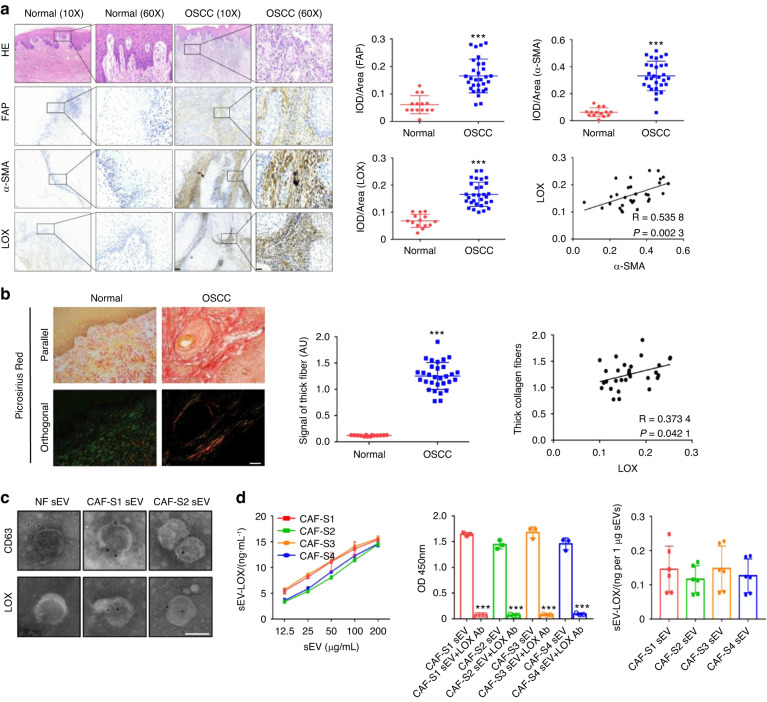
CAFs, collagen crosslinking, and LOX expression patterns in CAF sEV. **a** H&E and the expression of FAP, α-SMA and LOX in OSCC cases (*n* = 30) and normal controls (*n* = 14). Left, images of normal and OSCC tissues. (Scale bar = 100 μm, 20 μm). Right, quantification of FAP, α-SMA, LOX expression and the correlation of α-SMA with LOX expression in OSCC tissues. **b** Collagen crosslinking evaluation by picrosirius red staining in normal (*n* = 14) and OSCC stroma (*n* = 30). Left, images viewed under parallel and orthogonal polarizing filters. (Scale bar = 50 μm). Right, quantification of images viewed under orthogonal polarizing filters and the correlation of LOX expression with thick collagen fibers in OSCC stroma. **c** Immunogold labeling of CD63 and LOX on NF and CAF-S1/S2 sEV. (Scale bar = 100 nm). **d** ELISA examination of LOX in CAF-S1/S2/S3/S4 sEV (*n* = 3 per group). ***P < 0.001

To investigate CAF-induced collagen crosslinking in OSCC patients, 30 OSCC cases and 14 normal controls were collected (Table S1a). Correlation between the expression of LOX and the clinical parameters in OSCC patients were analyzed (Table S1b). Immunohistochemical staining identified higher expressions of fibroblast activation protein (FAP), α-smooth muscle actin (α-SMA) and LOX in the stroma of OSCC cases than normal controls. Positive significant association between the expression of LOX and α-SMA in the stroma of OSCC cases (Fig. 1a). Picrosirius red staining showed that the amount of fibrillar collagen increased markedly in OSCC stroma compared with normal controls. Positive significant association between the expression of LOX and thick fibers in the stroma of OSCC cases (Fig. 1b). These findings suggest that LOX derived from CAFs promote collagen crosslinking in the stroma of OSCC patients.

CAFs were isolated from the tissue samples of human OSCC cases. Normal fibroblasts (NFs) were isolated from human normal gingival tissues. The characteristics of CAFs and NFs were examined (Fig. S1). These cells express typical biomarkers of activated fibroblasts, including vimentin and fibroblast specific protein-1 (FSP-1) and do not express pan-cytokeratin, an epithelial biomarker. FAP and α-SMA were positive for CAFs and negative for NFs examined by immunofluorescence staining (Fig. S1a). At the same time, western blot showed that FAP and α-SMA were highly expressed in CAFs and slightly expressed in NFs (Fig. S1b). Conditioned medium (CM) collected from CAFs increased the proliferation and migration abilities of UM-SCC6 cells, compared to NF CM (Fig. S1c, d). LOX was highly expressed in CAFs and slightly expressed in NFs (Fig. S1a). The sEVs were isolated from the respective CM of UM-SCC6, CAF-S1/S2/S3/S4 and NF by differential ultracentrifugation (Fig. S2a) and showed the typical morphology of exosomes in transmission electron microscopy (TEM) images (Fig. S2b). They were positive for sEV biomarkers, including CD63, CD9, and HSP70, and negative for CALNEXIN, a biomarker of the endoplasmic reticulum (Fig. S2c). The size distribution and particle concentration of sEVs were measured using a nanoparticle tracking system. The average size of these sEVs was approximately 130 nm, and there were approximately 6 × 10^7^ sEVs per 1 mL CM (Fig. S2d). An immunogold labeling assay was performed to examine the association of LOX with CAF sEVs. CD63 served as the controls for sEV surface proteins. The results showed that CD63 and LOX proteins were present on the surface of CAF-S1/S2/S3/S4 sEVs, whereas αLOX was not present on the surface of NF sEVs (Fig. 1d, S3). The concentration of sEV-associated LOX (sEV-LOX) was determined by enzyme-linked immunosorbent assay (ELISA) using intact CAF-S1/S2/S3/S4 sEVs. The concentration of sEV-LOX in CAF-S1/S2/S3/S4 increased in a sEV concentration-dependent manner, and treatment with LOX blocking antibody significantly decreased the detectable sEV-LOX concentration. The concentration of CAF-S1/S2/S3/S4 sEV-LOX was approximately 0.15 ng·µg^−1^ sEVs (Fig. 1e). These findings suggest that LOX is located on the surface of CAF sEVs.

### CAF sEVs bind to the ECM and mediate collagen crosslinking

**Fig. 2:**
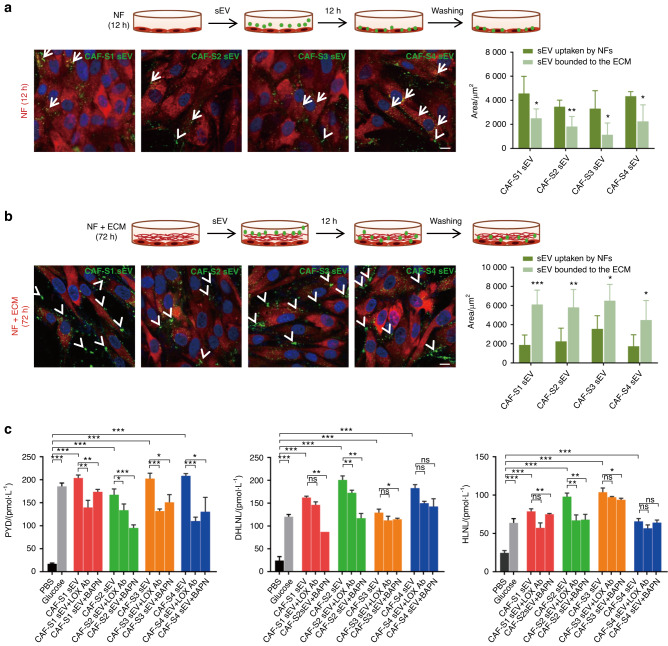
Collagen crosslinking triggered by CAF sEV-LOX. **a** Internalization of CAF-S1/S2/S3/S4 sEV (green) by cells. Normal fibroblasts (NFs) were cultured for 12 h (*n* = 3 per group). sEVs were mostly internalized into NFs (red) in large numbers (arrows). Small numbers of sEVs bound to the ECM (arrowheads). Left, representative images. (Scale bar = 10 µm). Right, quantification results. **b** NFs (red) were cultured for 72 h (*n* = 3 per group). Adhesion of CAF-S1/S2/S3/S4 sEV (green) to ECM (arrowheads). Left, representative images. (Scale bar = 10 µm). Right, quantification results. **c** ELISA assay of PYD, DHLNL, HLNL. NFs were cultured for 72 h, then treated with CAF-S1/S2/S3/S4 sEV with or without anti-LOX antibody or BAPN (*n* = 3 per group). PBS was used as a negative control (*n* = 3 per group) and glucose (*n* = 3 per group) was used as a positive control. *ns*, not significance, *P < 0.05, **P < 0.01, ***P < 0.001

To determine whether CAF sEVs interact with the ECM, two experiments were designed. In the first experiment, NFs were seeded in a 12-well plate and cultured for 12 h to allow cells to fully adhere to the wall while producing a small amount of ECM. Then, PKH67-labeled CAF sEVs were added into each well and cultured for another 12 h (Fig. 2a). In the second experiment, NFs were cultured for 72 h to induce the production of a large amount of ECM, followed by incubation with PKH67-labeled sEVs for 12 h (Fig. 2b). In the first experimental group, CAF-S1/S2/S3/S4 sEVs were internalized into NFs in large numbers (Fig. 2a). By contrast, in the second experimental group, CAF-S1/S2/S3/S4 sEVs were mostly located extracellularly and bound to the ECM instead of being uptaken by NFs (Fig. 2b). Compared to CAF sEVs, the affinity of OSCC-derived sEVs to ECM is low (Fig. S4). These findings indicate that CAF sEVs preferentially bind to the ECM when sufficient ECM is present.

Next, we analyzed the effect of CAF sEV binding on the ECM. NFs were cultured for 72 h and then incubated with CAF-S1/S2/S3/S4 sEVs for 12 h. To assess collagen crosslinking, we measured the levels of mature (PYD) and immature (DHLNL and HLNL) collagen crosslinks. PYD, DHLNL, and HLNL levels were significantly higher in the glucose and CAF-S1/S2/S3/S4 sEV groups than in the PBS-treated group (Fig. 2c). Treatment with the LOX inhibitor β-amino propionitrile (BAPN) or a LOX blocking antibody decreased the levels of PYD, DHLNL, and HLNL, especially the levels of the mature collagen crosslink PYD induced by the four types of CAF sEVs. These findings indicate that CAF sEVs induce collagen crosslinking, and CAF sEV-associated LOX plays an important role in this process.

### αLOX interacts with FN, POSTN, and BMP-1 on the surface of CAF sEVs

**Fig. 3:**
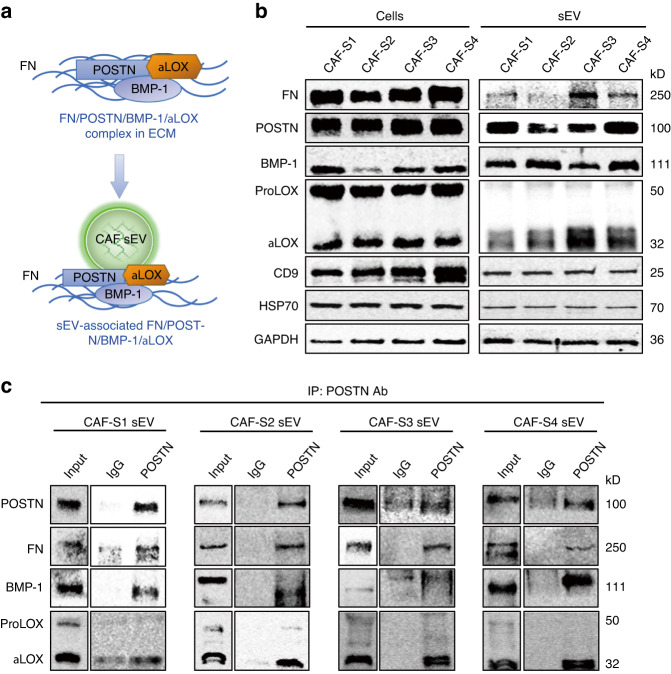
FN/POSTN/BMP-1/αLOX complex associated with CAF sEV. **a** Schematic of FN/POSTN/BMP-1/αLOX complex associated with sEV. **b** Western blot analysis of FN, POSTN, BMP-1 and LOX expression in CAF-S1/S2/S3/S4 and their sEVs. CD9 and HSP70 were used as CAF sEV markers. **c** IP examination of POSTN interaction with BMP-1 and αLOX in CAF-S1/S2/S3/S4 sEV. For blots source data, see Fig. S11

**Fig. 4:**
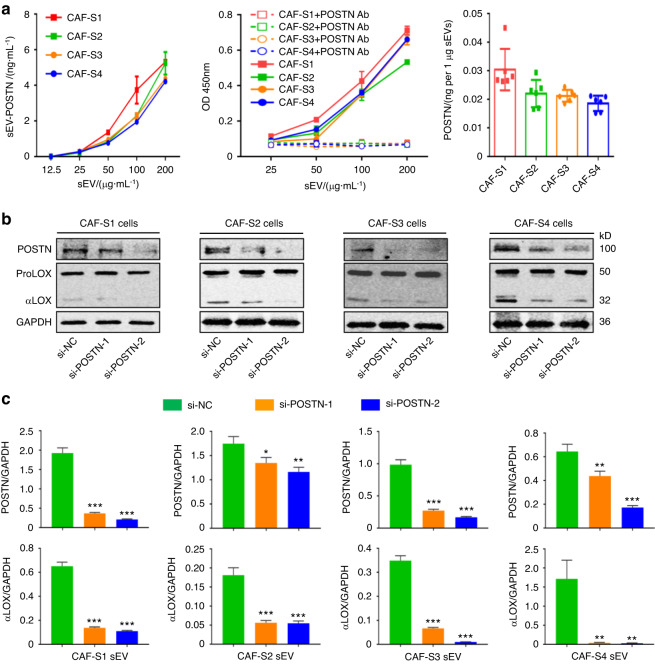
Regulation of αLOX by POSTN in CAF and their sEVs. **a** ELISA examination of POSTN in CAF-S1/S2/S3/S4 sEV (*n* = 3 per group). **b** Western blot analysis confirmed the downregulation of POSTN protein expression in CAF-S1/S2/S3/S4 induced by transfection with si-POSTN-1 or si-POSTN-2 compared with si-NC (*n* = 3 per group). **c** Quantitative analysis of POSTN and αLOX protein expression in CAF-S1/S2/S3/S4 sEV with POSTN downregulation (*n* = 3 per group). For blots source data, see Fig. S11. *P < 0.05, **P < 0.01, ***P < 0.001

We investigated how αLOX is located on the surface of CAF sEVs. POSTN binds to FN and recruits BMP-1 to the matrix, where BMP-1 cleaves pro-LOX to release αLOX. FN is one of the most extensively studied ECM molecules with respect to surface interaction with EVs.^38^ We hypothesized that αLOX may be associated with sEVs by FN (Fig. 3a). The expression of FN, POSTN, BMP-1 and αLOX in CAF-S1/S2/S3/S4 and their sEVs was confirmed by western blot (Fig. 3b). Immunoprecipitation assays confirmed that POSTN/FN interacted with BMP-1, and αLOX on CAF-S1/S2/S3/S4 (Fig. S5a) and their sEVs (Fig. 3c, S5b). The results of ELISA demonstrated that the levels of POSTN in CAF-S1/S2/S3/S4 sEVs increased significantly in a sEV concentration-dependent manner, and treatment with a POSTN-blocking antibody interfered with POSTN detection (Fig. 4a). CAF sEV-associated POSTN was approximately 0.02 ng per 1 µg sEVs. Next, CAF-S1/S2/S3/S4 were transfected with si-POSTN-1 and si-POSTN-2 to silence POSTN (Fig. 4b). si-POSTN-1 and si-POSTN-2 downregulated POSTN mRNA and protein expression in CAF-S1/S2/S3/S4 (Fig. 4b, S6a). Consistent with the cellular αLOX, POSTN knockdown decreased αLOX levels in sEVs isolated from the transfected CAF-S1/S2/S3/S4 (Fig. 4c, S6b). These findings suggest that generation of αLOX in CAFs and their sEVs was dependent on POSTN.

### CAF sEVs detect collagen I via surface integrin α2β1

**Fig. 5:**
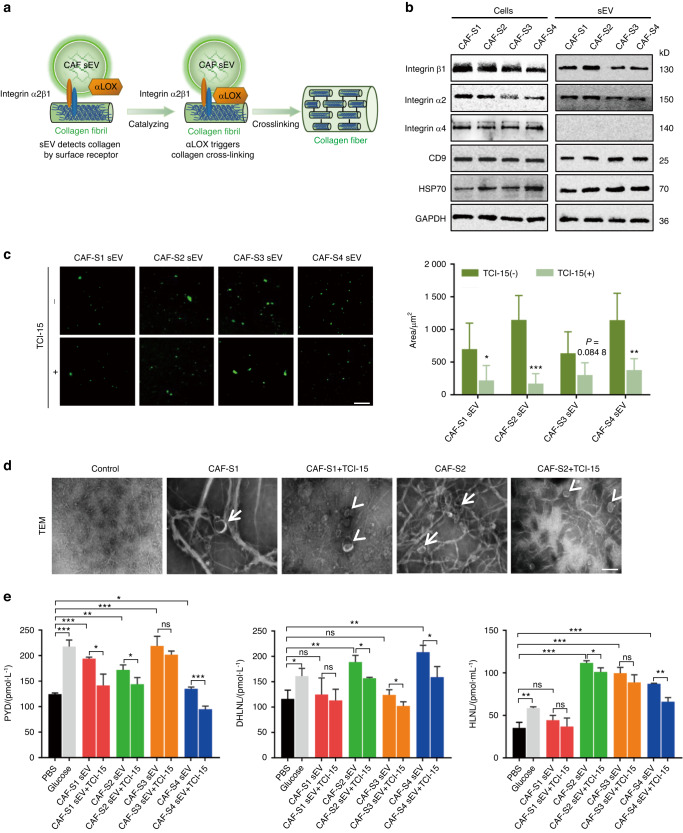
Collagen binding of CAF sEVs via integrin α2β1. **a** Schematic of collagen detection in CAF sEVs via the surface receptor. **b** Western blot analysis of integrin β1, integrin α2, and integrin α4 expression in CAF-S1/S2/S3/S4 and their sEVs. CD9 and HSP70 were used as CAF sEV markers. **c** Inhibition of the binding of CAF-S1/S2/S3/S4 sEV (green) to collagen I by treatment with TC I-15 in vitro (*n* = 3 per group). Left, representative images. (Scale bar = 50 µm). Right, quantification results. **d** Representative TEM images of collagen crosslinking induced by CAF-S1/S2 sEV with or without TC I-15 treatment. PBS was used as a control. Thick collagen fibers (arrows) and thin collagen fibers (arrowheads) associated with sEVs were observed. (Scale bar = 100 nm). **e** ELISA assay of PYD, DHLNL, HLNL levels in the collagen matrix treated with CAF-S1/S2/S3/S4 sEV with or without TC I-15 (*n* = 3 per group). For blots source data, see Fig. S11. *ns*, not significance, *P < 0.05, **P < 0.01, ***P < 0.001

Next, we analyzed the mechanisms by which CAF sEVs detect collagen. Integrin α2β1 is an important receptor involved in binding to collagen and activation of platelets. We hypothesized that integrin α2β1 may mediate the interactions between CAF sEVs and collagen (Fig. 5a). The presence of integrin α2 and β1 and the absence of integrin α4 in CAF-S1/S2/S3/S4 and their sEVs were confirmed by western blot (Fig. 5b). Incubation of collagen I matrix with PKH-67-labeled CAF-S1/S2/S3/S4 sEVs with or without TC I-15, an inhibitor of integrin α2β1, showed that a large amount of sEVs adhered to the collagen matrix, and TC I-15 suppressed the adhesion of CAF-S1/S2/S3/S4 sEVs to collagen significantly (Fig. 5c).

TEM was used to visualize collagen I with and without CAF sEV treatment for 12 h. Representative TEM images are shown in Fig. 5d, S7. In the absence of CAF-S1/S2/S3/S4 sEVs, the collagen I matrix appeared as thin, short, and curved fibers, whereas the CAF-S1/S2/S3/S4 sEV-treated group showed thick, long, and straight fibers. CAF-S1/S2/S3/S4 sEVs physically adhered to thick collagen fibers, and the collagen crosslinking induced by CAF-S1/S2/S3/S4 sEVs showed a diffuse pattern. Treatment with TC I-15 decreased the amount of thick and long fibers induced by CAF-S1/S2/S3/S4 sEVs. These findings indicate that CAF sEVs may detect collagen via integrin α2β1 and then directly induce collagen crosslinking catalyzed by CAF sEV-associated αLOX.

Next, we examined whether the collagen crosslinking promoted by CAF sEVs could be blocked by interfering with the binding of sEVs to collagen. The collagen matrix was treated with CAF-S1/S2/S3/S4 sEVs with or without TC I-15 for 12 h. PBS was used as a negative control and glucose was used as a positive control. The levels of mature and immature collagen crosslinks were measured by ELISA (Fig. 5e). The levels of PYD, DHLNL, and HLNL were significantly higher in the glucose and CAF-S1/S2/S3/S4 sEV groups than in the PBS group, and TC I-15 significantly inhibited CAF-S1/S2/S3/S4 sEV-induced upregulation of the mature collagen crosslink (PYD). The levels of immature collagen crosslinks (DHLNL and HLNL) were also lower in the CAF-S1/S2/S3/S4 sEV plus TC I-15 groups than in the CAF-S1/S2/S3/S4 sEV groups, although the difference was not consistently significant. These findings suggest that integrin α2β1 mediates the binding of CAF sEVs to collagen I.

### CAF sEV-αLOX causes EMT of OSCC

**Fig. 6:**
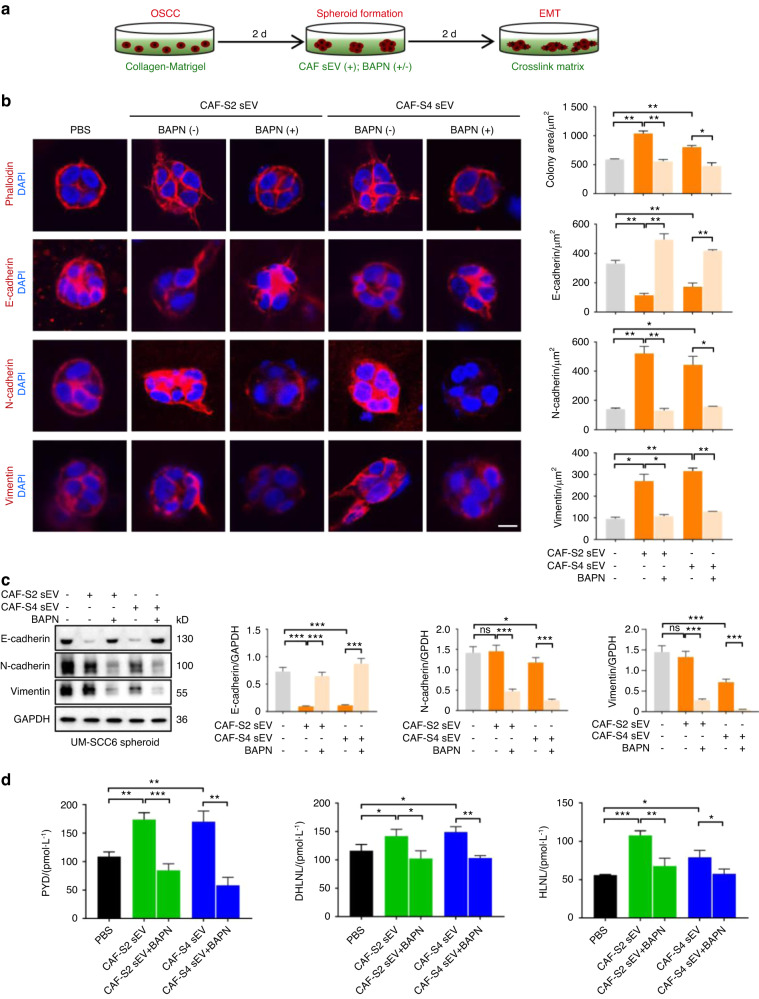
EMT of OSCC cells induced by CAF sEVs in vitro. **a** Illustration of collagen crosslinking in collagen I-Matrigel mixture triggered by CAF sEVs and drive EMT of OSCC. **b** The morphology (phalloidin) and expression of E-cadherin, N-cadherin and vimentin in UM-SCC6 spheroids stimulated by CAF-S2/S4 sEV with or without BAPN (*n* = 3 per group). PBS was used as a control (*n* = 3 per group). Left: representative images. (Scale bar = 10 µm). Right: quantitative analyses of cell invasion (phalloidin) and the expression of E-cadherin, N-cadherin and vimentin in UM-SCC6 spheroids under different experimental conditions (*n* = 3 per group). **c** Western blot analysis of E-cadherin, N-cadherin and vimentin in UM-SCC6 spheroids under different experimental conditions (*n* = 3 per group). **d** ELISA of PYD, DHLNL, HLNL in the collagen I-Matrigel matrix treated with CAF-S2/S4 sEV with or without BAPN (*n* = 3 per group). For blots source data, see Fig. S11. *ns*, not significance, *P < 0.05, **P < 0.01, ***P < 0.001

Collagen crosslinking causes ECM stiffness, which is a major regulator of EMT in cancer cells. We therefore examined the effect of CAF sEV-induced collagen crosslinking on OSCC cells in vitro. The experimental design is described in Fig. 6a. OSCC cells were seeded in a collagen-Matrigel mixture and cultured for 2 days. The tumor cells were evenly distributed in the matrix on day 0 and formed spheroids on day 2. The formed OSCC spheroids were stimulated with CAF-S2/S4 sEVs in the presence or absence of BAPN. PBS was used as the negative control. The morphology of the tumor spheroids was examined by staining cells with fluorescein phalloidin for F-actin and 4,6-diamidino-2-phenylindole (DAPI) for nuclei. As shown in Fig. 6b, UM-SCC6 spheroids were round and hardly invaded the surrounding matrix. By contrast, addition of CAF-S2/S4 sEVs into the culture medium caused the extension of long processes and invasion of UM-SCC6 cells into the surrounding matrix. The spheroid areas in the CAF-S2/S4 sEVs groups were significantly larger than those in the PBS group. BAPN treatment significantly reduced UM-SCC6 invasion induced by CAF-S2/S4 sEVs. Immunofluorescence detection of the EMT biomarkers E-cadherin, N-cadherin, vimentin in UM-SCC6 spheroids showed that CAF-S2/S4 sEVs groups significantly decreased E-cadherin expression and significantly increased N-cadherin, vimentin compared with the PBS group. BAPN rescued E-cadherin expression and downregulated N-cadherin and vimentin in UM-SCC6 spheroids. The protein levels of E-cadherin, N-cadherin, vimentin in UM-SCC6 and CAL-27 spheroids induced by CAF-S2/S4 sEVs significantly increased. BAPN decreased the protein levels of E-cadherin, N-cadherin, vimentin in UM-SCC6 (Fig. 6c) and CAL-27 spheroids (Fig. S9a). These finding suggesting that CAF sEVs promote OSCC EMT in the 3D matrix via αLOX.

ELISA was used to measure the levels of mature and immature collagen crosslinks in the collagen-Matrigel mixture (Fig. 6d). PYD, DHLNL, and HLNL levels increased significantly in the CAF-S2/S4 sEV groups compared with the PBS group, whereas BAPN treatment significantly decreased the levels of PYD, DHLNL, and HLNL induced by CAF-S2/S4 sEV. These findings suggest that CAF sEVs induce crosslinking of the collagen component in the collagen-Matrigel matrix and promote OSCC EMT.

**Fig. 7:**
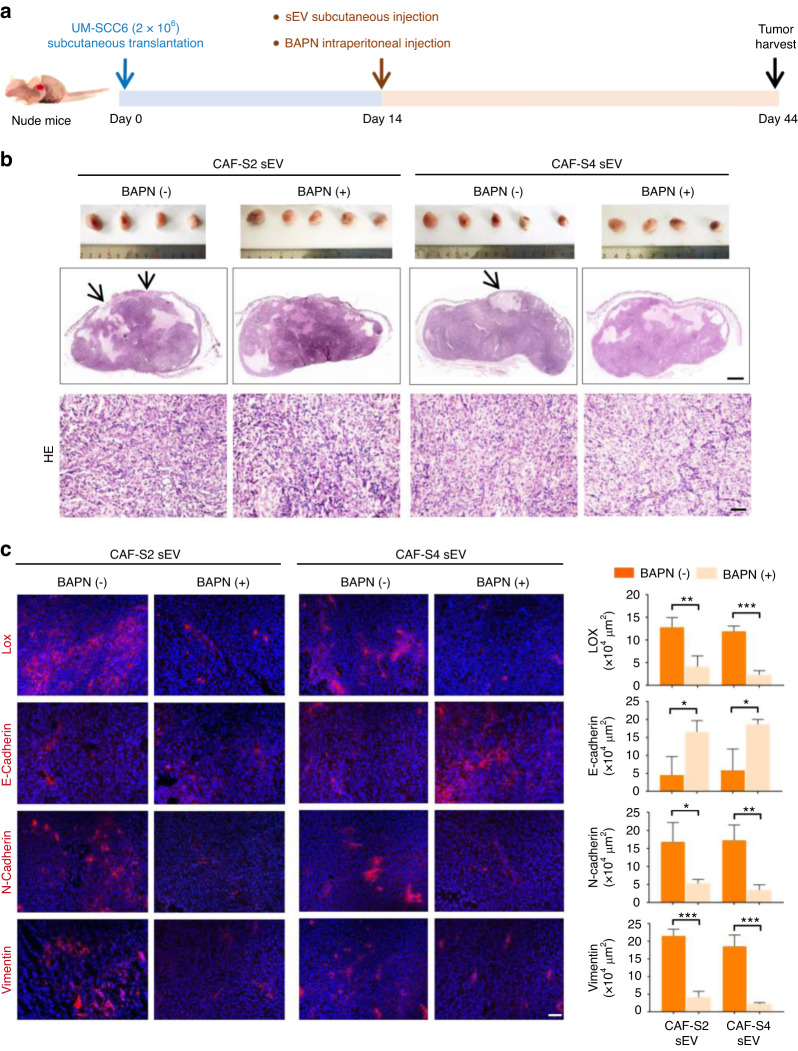
OSCC EMT induced by CAF sEVs in vivo. **a** Experimental steps. **b** Gross and histopathological examinations of UM-SCC6 xenografts treated with CAF-S2/S4 sEV with or without BAPN (*n* = 5 per group). (Scale bar = 100 µm). **c** Expression of LOX, E-cadherin, N-cadherin and vimentin in UM-SCC6 xenografts (*n* = 5 per group). Left, representative images. (Scale bar = 50 µm). Right, quantification results. *P < 0.05, **P < 0.01, ***P < 0.001

To determine the role of CAF sEV-αLOX in the EMT of OSCC in vivo, nude mice were subcutaneously injected with UM-SCC6 cells. When the average diameter of xenografts reached approximately 5 mm at 2 weeks, CAF sEVs were injected into the peritumor region every 3 days with or without BAPN intraperitoneal injection every other day (Fig. 7a). PBS was used as a control. The xenograft volume increased gradually in all groups (Fig. S10a). At 6 weeks, the mice were sacrificed and xenografts were harvested (Fig. 7b, S10b). The xenograft weight was higher in the CAF sEV groups than in the CAF sEV with BAPN groups, whereas the weight of the nude mice was comparable between the CAF-S2/S4 sEV and CAF-S2/S4 sEV with BAPN groups (Fig. S10c). The histopathological features of the xenografts were consistent with those of squamous cell carcinoma with low differentiation. Xenografts in the CAF sEV groups tended to invade the covering skin and formed ulcerations (Fig. 7b). BAPN significantly inhibited LOX expression in the xenografts (Fig. 7c). E-cadherin expression was higher, whereas N-cadherin and vimentin were lower in the CAF-S2/S4 sEV plus BAPN xenografts than in the CAF-S2/S4 sEV groups (Fig. 7c). These findings indicate that CAF sEV-αLOX may modulate EMT of OSCC in vivo.

### CAF sEV-αLOX promotes EMT of OSCC by activating YAP

**Fig. 8:**
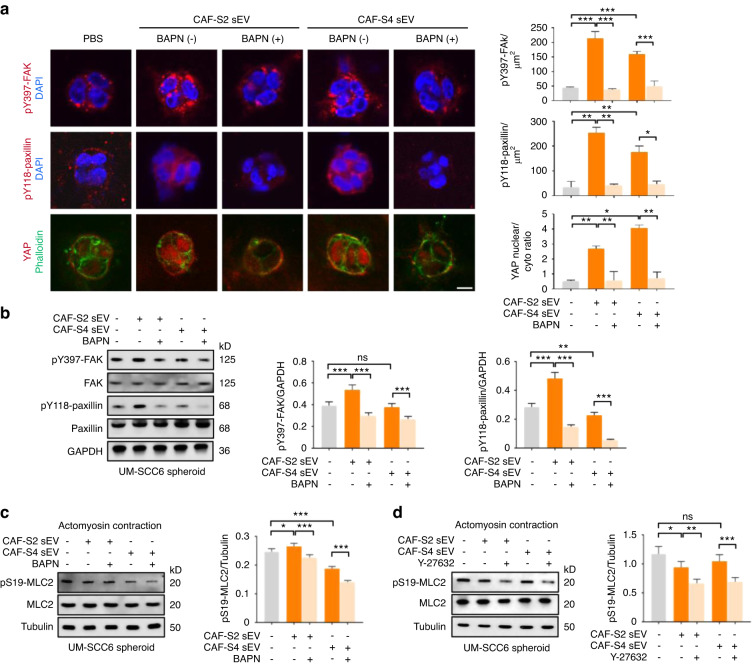
Crosslinked matrix activate p-FAK/p-paxillin/YAP signaling. **a** Expression of pY397-FAK, pY118-paxillin and YAP in UM-SCC6 spheroids stimulated by CAF-S2/S4 sEV with or without BAPN (*n* = 3 per group). PBS was used as a control (*n* = 3 per group). Left: representative images. (Scale bar = 10 µm). Right: quantification results. **b** Western blot analysis of the expression of pY397-FAK and pY118-paxillin in UM-SCC6 spheroids under different experimental conditions (*n* = 3 per group). **c** Expression of pS19-MLC2 in UM-SCC6 spheroids stimulated by CAF-S2/S4 sEV with or without BAPN (*n* = 3 per group). **d** Expression of pS19-MLC2 in UM-SCC6 spheroids stimulated by CAF-S2/S4 sEV with or without Y-27632 (*n* = 3 per group). For blots source data, see Fig. S11. *ns*, not significance, *P < 0.05, **P < 0.01, ***P < 0.001

Cells sense the changes of ECM via Hippo pathway and yes-associated protein (YAP) is the core component of the Hippo pathway.^35^ Increased adhesive area caused by collagen crosslinking may promote YAP nuclear localization and target gene induction. We next investigated whether the EMT of OSCC cells stimulated by CAF sEVs was promoted by YAP activation. It was found that UM-SCC6 spheroids in the CAF-S2/S4 sEV-treated groups showed YAP nuclear translocation and high expression of pY397-FAK and pY118-paxillin, compared with those in the PBS groups (Fig. 8a). Moreover, BAPN hindered YAP nuclear location and high expression of pY397-FAK and pY118-paxillin in UM-SCC6 spheroids stimulated by CAF-S2/S4 sEVs (Fig. 8a). Similarly, western blot analysis demonstrated CAF-S2/S4 sEVs promoted the expression of pY397-FAK and pY118-paxillin in both UM-SCC6 (Fig. 8b) and CAL-27 spheroids (Fig. S9b). And BAPN decreased the expression of pY397-FAK and pY118-paxillin. To confirm whether contractility of actomyosin cytoskeleton was involved in YAP nuclear translocation, the main regulator of actomyosin contraction, phosphor-S19-myosin light chain 2 (pS19-MLC2), was examined. It was found that the expression of pS19-MLC2 in both UM-SCC6 and CAL-27 groups treated with CAF-S2/S4 sEVs significantly increased, compared with those treated with CAF-S2/S4 plus BAPN (Fig. 8c, S9c). Furthermore, ROCK inhibitor Y-27632 decreased the expression level of pS19-MLC2 (Fig. 8d, S9d), suggesting the role of contractility of actomyosin cytoskeleton in YAP activation. We also examine the in vivo expression of pY397-FAK, pY118-paxillin and YAP in OSCC cells. It was found that UM-SCC6 cells showed significantly higher expression of pY397-FAK, pY118-paxillin and YAP in the CAF-S2/S4 sEV groups than those in the CAF-S2/S4 sEV plus BAPN group (Fig. S8). These findings indicate that CAF sEV-αLOX may modulate EMT of OSCC through FAK/paxillin/YAP pathway.

**Fig. 9:**
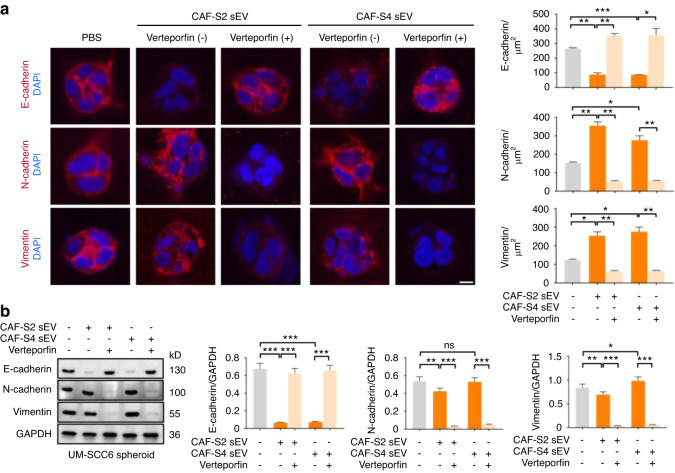
YAP modulate EMT of OSCC cells induced by CAF sEVs in vitro. **a** Expression of E-cadherin, N-cadherin and vimentin in UM-SCC6 spheroids stimulated with CAF-S2/S4 sEV with or without verteporfin (*n* = 3 per group). PBS was used as a control (*n* = 3 per group). Left: representative images. (Scale bar = 10 µm). Right: quantitative analyses of the expression of E-cadherin, N-cadherin and vimentin in UM-SCC6 spheroids under different experimental conditions (*n* = 3 per group). **b** Western blot analysis of the expression of E-cadherin, N-cadherin and vimentin in UM-SCC6 spheroids under different experimental conditions (*n* = 3 per group). For blots source data, see Fig. S11. *ns*, not significance, *P < 0.05, **P < 0.01, ***P < 0.001

Next we investigated the effects of verteporfin on inhibiting OSCC EMT. It was found that verteporfin significantly decreased the expression of E-cadherin, N-cadherin, vimentin in UM-SCC6 spheroids induced by CAF-S2/S4 sEVs (Fig. 9a). Similarly, western blot analysis demonstrated that verteporfin significantly decreased the expression levels of E-cadherin, N-cadherin, and vimentin in both UM-SCC6 (Fig. 9b) and CAL-27 spheroids (Fig. S9e).

## Discussion

**Fig. 10:**
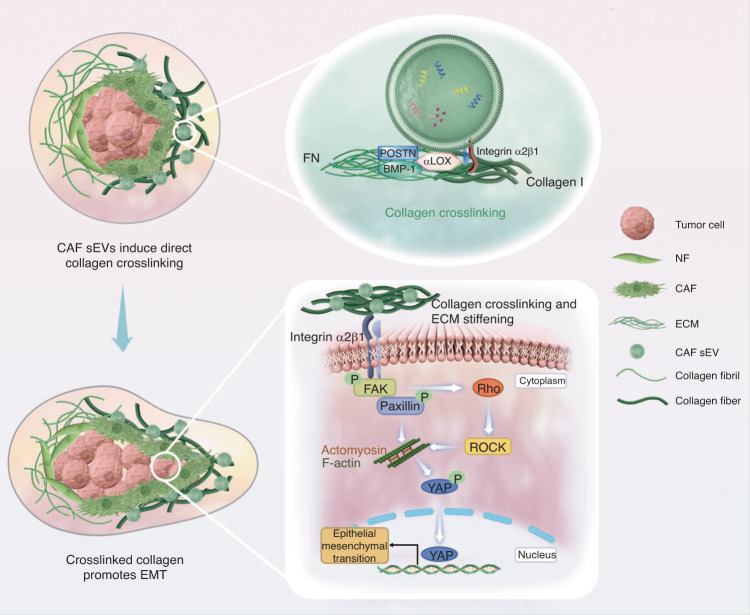
Schematic of collagen crosslinking triggered by CAF sEVs leading to EMT of OSCC. CAFs in the tumor microenvironment secrete sEVs enriched in α-LOX, which interact with FN, POSTN, and BMP-1. CAF sEVs detect collagen via integrin α2β1 and induce direct collagen crosslinking. The crosslinked matrix activates p-FAK/p-paxillin pathway. Rho/ROCK regulates actomyosin contraction, and then leads to the nuclear translocation of YAP, which in turn promotes EMT of OSCC cells

Collagen is the most abundant ECM scaffolding protein, and enhanced collagen crosslinking is associated with tumor progression.^7,39^ In this study, we demonstrated that sEVs derived from CAFs mediate collagen crosslinking directly. CAFs secrete αLOX-rich sEVs that detect collagen I via surface integrin α2β1 and subsequently induce collagen crosslinking, and collagen crosslinking promotes EMT in OSCC via p-FAK/p-paxillin/YAP signaling (Fig. 10).

ProLOX and αLOX are generated by processing full-length LOX. BMP-1 catalyzes the extracellular proteolytic cleavage of proLOX to release αLOX and a propeptide.^21,40^ αLOX plays a critical role in the formation and repair of the ECM by catalyzing the crosslinking of collagen and elastin, which stabilizes these fibrous proteins.^41,42^ In this study, both proLOX and αLOX were detected in CAF cells, suggesting that at least a part of αLOX is generated in CAFs. The difference between the present results and those of previous studies might be due to the use of stromal cells in this study, whereas previous studies used various cancer cells. We demonstrated that CAFs express BMP-1, which cleaves proLOX, whereas most cancer cells do not express BMP-1 and need exogenous BMP-1 to catalyze the processing of proLOX secreted into the extracellular space. We showed that only αLOX, but not proLOX, was associated with CAF sEVs. We further demonstrated that αLOX interacts with POSTN, FN, and BMP-1 on the surface of CAF sEVs and is catalytically active, as indicated by its effect on collagen crosslinking. These findings suggest that CAF sEVs not only carry αLOX, but may also be responsible for the production of αLOX from proLOX catalyzed by the FN-POSTN-BMP-1 complex. The present findings provide new insight into the processing of proLOX into αLOX in stromal cells.

LOX is commonly known as a secreted protein, but not a membrane protein. It has been demonstrated that FN is captured on the surface of EVs by integrins or heparan sulfate proteoglycans.^38,43^ We demonstrated that αLOX is located on the surface of CAF sEVs by interacting with FN, POSTN and BMP-1. It has been demonstrated that proLOX binds to FN is critical for proteolytic activation of LOX.^44^ The present study showed that overexpression of POSTN promoted the proteolytic cleavage of the propeptide, which increased the amount of αLOX in calvarial osteoblasts cells.^24^ Our study demonstrated that when POSTN function is normal, both proLOX and αLOX can be detected in CAF cells, while CAF sEVs only carry αLOX. We further demonstrated that αLOX interacted with FN, POSTN, and BMP-1 on the surface of CAF sEVs. The functions of EV-associated FN include facilitating cellular uptake of EVs, promoting directional cancer cell movement, and inducing IL-1β expression by macrophages. We further demonstrate that EV-associated FN may serve as a reservoir for some soluble factors, which could not bind to EV directly.

EVs play important roles in cell-cell communication by delivering their cargo to recipient cells.^45–48^ However, limited information is available regarding the interaction between EVs and the ECM. A recent study identified several proteolytic proteins associated with EVs that may cleave ECM components.^49^ Membrane type 1 matrix metalloproteinase (MT1-MMP) is an efficient ECM degrading enzyme. Functionally active MT1-MMP could be secreted from EVs and degrade type I collagen and gelatin.^50^ Activated polymorphonuclear leukocytes secrete EVs with high levels of surface-bound neutrophil elastase. These EVs bind to the ECM via integrin MAC-1 and degrade collagen I via neutrophil elastase. When administered to mice, these EVs cause emphysema in animal models.^51^ In this study, we found that CAFs released sEVs containing high levels of surface-bound αLOX. We showed that CAF sEVs bind to collagen I via integrin α2β1 and promote crosslinking of collagen directly via sEV-LOX. These findings expand the biological functions of EVs and highlight the important biological effects of these particles on both cells and the ECM.

Collagen crosslinking causes ECM stiffening changes the mechanical stress microenvironment of cells and activated YAP signaling regulated by the non-canonical Hippo mechanotransduction pathway.^52^ Matrix stiffness activates YAP in vascular endothelial cell through cytoskeletal reorganization, and promotes tip cell formation during angiogenesis.^53^ YAP activation causes the nuclear/cytoplasmic translocation, which binds to the TEAD family of transcription factors and serve to activate the expression of pro-proliferative and survival enhancing EMT of tumor cells.^54–56^ Mechanotransduction leads to the increased contractility of actomyosin cytoskeleton, reflected by increased ECM stiffness. Then, actomyosin contraction with increased tension leads to nuclear translocation of YAP and subsequently mechanosensing is linked to activity of nuclear transcription factors, thereby promotes EMT in tumor.

LOX directly affects tumor progression by regulating collagen crosslinking and the stiffness of the ECM, which are factors that modulate cell invasion and tumor progression.^7,57^

LOX expression in patients with head and neck squamous cell carcinoma decreases metastasis-free and overall survival.^58^ CAF-derived LOX in the liver metastatic niche of gastric cancer promotes tumor cell proliferation and outgrowth.^59^ Stromal fibrosis accompanied by collagen crosslinking is common in OSCC. Recently, a study demonstrated that CAF-derived LOX promotes invasion and EMT process in OSCC cells by activating FAK phosphorylation pathway.^60^ In this study, we focused on the interaction between CAF sEVs and ECM and found that CAF sEV-αLOX stimulated collagen crosslinking directly. Furthermore, OSCC cells sensed the altered ECM and led to the activation FAK/paxillin/ROCK pathway and YAP nuclear translocation. High activity of YAP in turn stimulated EMT of OSCC. These findings reveal a direct role of CAF sEV-associated LOX in collagen crosslinking and offer a new paradigm for understanding the positive association between CAFs in the stroma and increased invasive ability of OSCC.

In conclusion, CAF-released sEVs were rich in αLOX, which interacted with FN, POSTN, and BMP-1 on the surface of sEVs. Integrin α2β1 mediated the binding of CAF sEVs to collagen I, and αLOX triggered collagen crosslinking directly. Collagen crosslinking induced by CAF sEVs promoted the EMT of OSCC via FAK/paxillin/ROCK/YAP pathway. These findings elucidate a critical mechanism underlying tumor ECM alteration and reveal a novel role of sEVs in ECM remodeling and its effect on cancer cells.

## Materials and methods

### Ethics approval and consent to participate

The use of human samples was approved by the Ethics Committee of Dalian Medical University. Animal tests were conducted in compliance with the guidelines issued by the Ethical Committee of Dalian Medical University (No. AEE20017).

### Immunohistochemical staining

Paraffin-embedded tumor tissues from 30 patients with OSCC undergoing surgical resection were studied. Hematoxylin and Eosin (H&E) staining was used to confirm the diagnosis. The clinical and histopathological features of these OSCC cases were shown in Table S1. 14 cases of normal oral mucosa tissues were used as controls. Immunohistochemical staining was performed using SPlink Detection Kits (SP-9000, ZSGB-BIO, China). The sections were deparaffinized and rehydrated. Endogenous peroxidase activity was blocked with 3% hydrogen peroxide in methanol. Nonspecific binding sites were blocked with 10% goat serum. Sections were incubated antibodies against FAP (1:200; Abcam, Cambridgeshire, UK), α-SMA (1:100; Proteintech, Wuhan, China) or LOX (1:100; Abcam) overnight at 4 °C. Immunoreactions were detected using 3, 3ʹ-diaminobenzidine as the final chromogen. Nuclei were counterstained with hematoxylin. Negative control experiments were carried out by replacing the primary antibody with PBS. The integrated optical density and area of target distribution were measured with Image-Pro® Plus version 6.0. We calculated the mean density of each field, and took the average of the mean density of at least 10 fields as the mean density of each case.

### Picrosirius red staining and quantification

In total, 4-µm-thickness paraffin sections were prepared and stained with 0.1% picrosirius red (Abcam) and counterstained with Weigert’s hematoxylin. Polarized lighting images were performed on a fluorescence microscope (Olympus BX43, Tokyo, Japan) fitted with an analyzer (U-ANT, Olympus) and polarized (U-POT, Olympus). Quantification of thick collagen fibers were performed using ten high power fields per case.

### Cell culture

Four cases of fresh OSCC tissues were obtained from the First and Second Affiliated Hospitals, Dalian Medical University (No. 2022-011). CAFs were isolated from OSCC tissues and named CAF-S1, CAF-S2, CAF-S3 and CAF-S4, respectively.^45^ NFs were isolated from normal gingival tissues of a healthy adult during tooth extraction. CAFs and NFs were cultured in DMEM/F12 medium (Gibco, Grand Island, NY, USA). UM-SCC6 was a kind gift from Peking Union Medical University and cultured in DMEM/High Glucose medium (Hyclone, Logan, UT, USA). CAL-27 was purchased from CELLCOOK and grown in DMEM/High Glucose medium (Hyclone). All cells were supplemented with 10% fetal bovine serum (FBS, ScienCell, Carlsbad, CA, USA), 100 U·mL^−1^ penicillin and 100 U·mL^−1^ streptomycin (Hyclone) at 37 °C in a humidified 5% CO_2_ incubator.

### Immunofluorescence staining

We used the fifth passage of primary cells for initial characterization by immunofluorescent staining. Cells were fixed with 4% paraformaldehyde for 20 min and permeabilized with PBS containing 0.025% Triton X-100. Samples were blocking with normal goat serum contained 5% bovine serum albumin (BSA) for 1 h, incubated with primary antibodies at 4°C overnight. The following primary antibodies were used: pan-cytokeratin (1:100; Merck Millipore, Temecula, CA, USA), vimentin (1:200; Abcam), FSP-1 (1:100; Abcam), FAP (1:100; Abcam), α-SMA (1:100; Proteintech) and LOX (1:100; Abcam). Then samples were treated with Dylight 549-Conjugated secondary antibody (1:200; Abbkine, Wuhan, China). Nuclei were counterstained with DAPI (1:3 000; ThermoFisher Scientific, Waltham, MA, USA). Images were recorded using an inverted microscope (Olympus IX71, Tokyo, Japan).

### sEV separation and labeling

Bovine sEVs were depleted from FBS by ultracentrifugation. When cells reached 80% confluency, fresh DMEM/F12 medium with 2% sEV-depleted FBS was used to culture cells for another 72 h as CM. Then, CM was collected and differentially centrifuged 500 × *g* for 10 min, 2 500 × *g* for 20 min, 12 000 × *g* for 30 min and 100 000 × *g* for 70 min. Then, the pellet was diluted in 20 mL PBS and ultracentrifuged at 100 000 × *g* for 70 min. The protein concentration of sEVs was measured by BCA kit (Beyotime Biotechnology, Shanghai, China). sEVs were labeled with PKH67 (Sigma-Aldrich, Louis, MO, USA) according to the company’s instruction. Labeled sEVs were re-separated by ultracentrifugation at 100 000 × *g* for 70 min.

### TEM and immunogold labeling

sEVs in PBS were placed on a formvar carbon-coated grid for 20 min. The sample was negatively stained with 1% phosphotungstic acid solution for 5 min. For immunogold labeling, purified sEVs in PBS were placed on formvar carbon-coated grid for 20 min, washed with PBS twice and incubated 50 mmol·L^−1^ glycine to quench free aldehyde groups. After the grids were washed, they were blocked using PBS containing 5% BSA for 1 h, and incubated with primary antibodies against CD63 (10 µg·mL^−1^, Abcam), LOX (10 µg·mL^−1^, Abcam) overnight at 4 °C. Then, the samples were incubated with goat anti-rabbit IgG conjugated to 5 nm-colloidal gold particles (ThermoFisher Scientific). Then the grid was washed with PBS contained 0.1% BSA and fixed with 2.5% glutaraldehyde for 15 min then thoroughly washed with deionized water. Samples were counterstained with phosphato-tungstic acid for 5 min. Images were taken using a transmission electron microscope (JEM-2000EX*, Japan Electronics, Japan).

### ELISA

To detect LOX or POSTN in sEVs, ELISA kits was purchased (LOX: Elabscience #H0174c; POSTN: Elabscience #H2452c). sEVs separated from CM were prepared for the serial dilution were added into each well (100 µL per well). After 90 min, biotinylated LOX or POSTN antibody was added into each well and incubated at 37 °C for 1 h. Then, horseradish peroxidase-conjugated streptavidin (100 µL) was added and incubated at 37 °C for 30 min followed by incubation with 3,3',5,5'-tetramethylbenzidine substrate reagent at 37 °C for 15 min. After adding 50 µL of stop solution to each well, the absorbance at 450 nm was measured with a microplate reader (ThermoFisher Scientific). To block the sEV surface LOX or POSTN, LOX or POSTN blocking antibodies (10 µg·mL^−1^) in 100 µL PBS were used to incubate with sEVs at 4 °C overnight, washed with PBS (20 mL), and pelleted by ultracentrifugation to remove non-bound antibodies. Each experiment was repeated at least three times.

### sEV treatment of human NFs

To assess the interactions of CAF sEVs with stromal cells and ECM, NFs (1 × 10^5^ cells per well) labeled with CellTracker Red CMTPX Dye (ThermoFisher Scientific) were cultured in a 12-well plate for 12 h or 72 h. Then, PKH67-labeled sEVs (20 μg per well) were added into each well and incubated for 12 h. After washing with PBS, NFs were fixed with 4% paraformaldehyde for 15 min. Nuclei were counterstained with DAPI (ThermoFisher Scientific). Images were recorded by an inverted microscope (Olympus IX71). To test collagen cross-linking, NFs (1 × 10^5^ cells per well) were seeded in a 6-well plate and cultured for 72 h. Purified sEVs (100 µg per well) were added into each well and incubated for 12 h. For LOX inhibition, sEVs (100 µg) were pre-incubated with 200 µmol·L^−1^ BAPN (Sigma-Aldrich) and the LOX-blocking antibody (10 µg·mL^−1^) at 4 °C overnight. The immature crosslinks, DHLNL and HLNL, and a mature crosslink, PYD, were quantified by ELISA assay. ELISA kits (DHLNL: Jonln #JL47954, HLNL: Jonln #JL47940, PYD: Jonln #JL19698) were purchased from Shanghai JONLN Reagent Co., Ltd., China. The absorbance at 450 nm was measured using a microplate reader (ThermoFisher Scientific).

### sEV treatment of human collagen matrix

To confirm sEVs bond to collagen matrix, type I collagen (3 mg·mL^−1^, Corning, Bedford, MA, USA) was mixed with Matrigel (Corning) at ratio 2:1 and added to a 48-well plate and incubated for 30 min at 37 °C. Then PKH67-labeled sEVs (20 µg per well) were added into the collagen I-containing wells and incubated at 37 °C for 12 h. To inhibit integrin α2β1-mediated collagen binding, sEVs were pre-incubated with 2 µM TCI-15 (TOCRIS, Oxfordshire, UK) at 37 °C for 1 h. After washing with PBS, images were recorded by an inverted fluorescent microscope (Olympus IX71). To visualize sEV-stimulated collagen crosslinking, collagen I (5 µg) was mixed with 20 µg sEVs and incubated for 12 h at 4 °C. PBS was used as a control. The samples were placed on a formvar carbon-coated grid for 20 min, then negatively stained with 1% phosphotungstic acid solution for 5 min. Any excess of stain was wicked away and grid was air-dried at room temperature. Images were recorded using a transmission electron microscope (JEM-2000EX*). To detect collagen crosslinks, sEVs (100 µg) treated by with and without TCI-15 (2 µmol·L^−1^) in a 24-well plate and incubated for 12 h. To detect collagen crosslinks in collagen I-Matrigel mixture (75 µL per well), sEVs (100 µg) treated by with and without BAPN (200 µmol·L^−1^) in a 96-well plate and incubated for 12 h. PYD, DHLNL, and HLNL were quantified by ELISA assay.

### Western blot analysis

Cells or sEVs were lysed by RIPA buffer (Millipore Corporation, Billerica, MA, USA) supplemented with Protease inhibitor cocktail (100 mmol·L^−1^, Solarbio, Beijing, China) and Phosphatase inhibitor cocktail (Solarbio) at 4 °C for 15 min. To extract the protein of UM-SCC6 and CAL-27 spheroids, each sample was added in RIPA buffer containing 3 mm-grinding beads and sequentially separated using a grinding machine (Servicebio, Wuhan, China). Protein concentration was determined by BCA kit (Beyotime Biotechnology). Equal amount of proteins was separated by sodium dodecyl sulfate-polyacrylamide gel. Proteins on gel were transferred onto a nitrocellulose membrane (Millipore Corporation). Then, the membrane was blocked with 5% fat-free milk for 2 h followed by primary antibodies incubation at 4°C overnight. Primary antibodies included CD63 (1:500; Abcam), CD9 (1:500; Abcam), HSP70 (1:500; Abcam), CALNEXIN (1:500; Proteintech), LOX (1:600; Abcam), POSTN (1:500; Abcam), BMP-1 (1:300; Bioss, Beijing, China), FN (1:500; Abcam), integrin α2 (1:500, Abcam), integrin α4 (1:500; Abcam), integrin β1 (1:500; Abcam), pY397-FAK (1:500; Abcam), FAK (1:1 000; Abcam), pY118-paxillin (1:500; Abcam), Paxillin (1:1 000; Abcam), pS19-MLC2 (1:1 000; Cell Signaling Technology, Danvers, MA, USA), MLC2 (1:1 000; Cell Signaling Technology), Tubulin (1:10 000; Absin, Shanghai, China), E-cadherin (1:1 000; Abcam), N-cadherin (1:1 000; Abcam), vimentin (1:1 000; Abcam), FSP-1 (1:1 000; Abcam), FAP (1:500; Abcam), α-SMA (1:1 000; Proteintech) and GAPDH (1:3 000; Proteintech). After the membrane incubation with horseradish peroxidase (HRP)-labeled IgG (H + L) as secondary antibody (1:3 000; Proteintech) for 1 h. Protein bands were detected with Enhanced chemiluminescence detection system (ChemiDoc XRS, Bio-Rad).

### RNA isolation and quantitative reverse transcription-polymerase chain reaction (qRT-PCR)

Total RNA was extracted using Trizol Reagent (ThermoFisher Scientific). The PrimerScript RT reagent Kit (TaKaRa, Dalian, China) was used for reverse transcription. Quantitative real-time PCR reaction was performed with SYBR Premix Ex Taq reagent kit (TaKaRa) using Thermal Cycler Dice Real Time System (TaKaRa). GAPDH was used as an internal control. The primers for POSTN and GAPDH were purchased from TaKaRa. Primer sequences were as following: POSTN (forward): 5'-CCA TCA CAT CGG ACA TAT TGG A-3', (reverse): 5'-TGC TCC TCC CAT AAT AGA CTC A-3'; GAPDH (forward): 5'-GTG AAG GTC GGA GTC AAC G-3', (reverse): 5'-TGA GGT CAA TGA AGG GGT C-3'. Each experiment was repeated at least three times.

### Immunoprecipitation

Protein A/G plus-magnetic beads (SelleckChem, Shanghai, China) was incubated with anti-POSTN antibody (Santa Cruz Biotechnology, Dallas, Texas, USA) or anti-FN antibody (Proteintech) under rotation for 15 min, followed by incubating with the protein extract from cells or sEVs for 15 min. The immunoprecipitated proteins were washed and resuspended in loading buffer, denatured and submitted to western blot analysis.

### siRNA transfection

Two POSTN gene-specific short interfering RNA (siPOSTN-1: 5' - CCC AUG GAG AGC CAA UUA UTT-3' and siPOSTN-2: 5'-CUC UGA CAU CAU GAC AAC AAA UTT-3') and a negative control siRNA (si-NC: 5'-UUC UCC GAA CGU GUC ACG UTT-3') were synthesized by Genepharma (Shanghai, China). They were transfected into cells with Lipofectamine™ 2000 reagent (ThermoFisher Scientific) according to manufacturer’s instructions. After 48 h, total RNA and protein were isolated from transfected cells.

### Preparation of tumor spheroid, immunostaining and imaging analysis

Collagen I (2 mg·mL^−1^) was mixed with Matrigel in the ratio 2: 1 by volume. A 96-well plate was pre-coated with the collagen I-Matrigel mixture (30 µL per well). UM-SCC6 or CAL-27 cells were suspended in the collagen I-Matrigel mixture and added to the precoated 96-well plate (3.75 × 10^4^ cells in 75 µL per well). After the mixture gelled, DMEM/High Glucose medium (100 µL per well) was added into each well. After 2 days, tumor cells formed spheroids in the wells and CAF sEVs (100 µg) were added into each well with or without BAPN (200 µmol·L^−1^). PBS was used as a control. To confirm YAP signaling, tumor spheroids in the wells and CAF sEVs (100 µg) with or without verteporfin (10 µmol·L^−1^) were added into each well. To investigate Rho/ROCK modulate actomyosin contraction, tumor spheroids in the wells and CAF sEVs (100 µg) with or without Y-27632 (10 µmol·L^−1^) were added into each well. After another 2 days, tumor spheroids were fixed with 4% paraformaldehyde for 15 min and permeabilized with PBS containing 0.025% Triton X-100. Samples were blocked using normal goat serum containing 5% BSA for 2 h at room temperature, then incubated with primary antibodies, including E-cadherin (1:100; Abcam), N-cadherin (1:100; Abcam), vimentin (1:100; Abcam), pY397-FAK (1:100; Abcam), pY118-paxillin (1:100; Abcam) and YAP (1:50; Santa Cruz Biotechnology) overnight at 4°C. Next day the samples were incubated with DyLight 549-conjugated secondary antibody (1:200; Abbkine) and nuclei were counterstained with DAPI (1:3 000; ThermoFisher Scientific). Phalloidin-TRITC (1:200; YEASEN, Shanghai, China) staining was performed to observed cellular morphologies. Phalloidin-FITC (1:200; Servicebio) was added to the samples before washing and image acquisition.

Images were recorded using a Nikon confocal microscope (Nikon A1R). UM-SCC6 spheroid area and expression areas of E-cadherin, N-cadherin, vimentin, pY397-FAK, pY118-paxillin and YAP were quantified with Image Pro Plus 6.0 using 5 random high-power fields of each well. The quantification of the YAP nuclear/cytoplasmic ratio was measured as previously described.^61,62^ Briefly, we calculated for each tumor spheroid the nuclear YAP intensity (region delimited by the DAPI staining) and the cytoplasmic YAP intensity (region delimited by Phalloidin-FITC staining excluding the nuclear region) for at least 10 tumor spheroids for each sample.

### Animal models

UM-SCC6 cells (2 × 10^6^ cells per mouse) were injected into the subcutaneous space of nude mice aged 6 weeks old (about 18 g, female). After 2 weeks, the average diameter of subcutaneous xenografts was around 5 mm. CAF sEVs (100 µg per mouse) were injected into the peritumor region every 3 days. BAPN (100 mg·kg^−1^) was injected intraperitoneally every other day. PBS was used as a control. Mice were weighed every 3 days. Xenografts were measured using calipers every week and the volumes were calculated by the formula: (width)^2^ × length/2. When most xenografts reached 1 000 mm^3^ at week 6, mice were sacrificed. Xenografts were harvested and fixed in 10% formalin buffer for 24 h, embedded in paraffin. H&E staining was performed to confirm the histological features of these xenografts. Immunofluorescent staining was performed to assess EMT and YAP signaling. Primary antibodies included LOX (1:200; Abcam), E-cadherin (1:100; Abcam), N-cadherin (1:100; Abcam), vimentin (1:100; Abcam), pY397-FAK (1:100; Abcam), pY118-paxillin (1:100; Abcam), YAP (1:50; Santa Cruz Biotechnology). All animal experiments were strictly performed in accordance with animal care guidelines approved by the Animal care and Use Committee of Dalian Medical University.

### Statistical analyses

GraphPad Prism 7.0 (Graphpad Software Inc.), Image Pro Plus 6.0 and Image J software were used for statistical analyses. Unpaired Student’s *t* test was performed for comparisons between all of the data. Correlation was analyzed by using Spearman test. Error bars shown in graphical data represent the mean ± SD. Statistical significance was defined as *P < 0.05. Each experiment was repeated at least three times.

## Supplementary information

Supplementary figure

Supplementary methods

Supplementary table
