# Supplementary material for: Carcinoma-associated fibroblast-derived lysyl oxidase-rich extracellular vesicles mediate collagen crosslinking and promote epithelial-mesenchymal transition via p-FAK/p-paxillin/YAP signaling
Source: Int J Oral Sci. 2023 Aug 2;15:32. doi: 10.1038/s41368-023-00236-1 (PMC10397209; doi:10.1038/s41368-023-00236-1)

Carcinoma-associated fibroblast-derived lysyl oxidase-rich extracellular vesicles mediate collagen crosslinking and promote epithelial-mesenchymal transition via p-FAK/p-paxillin/YAP signaling

Xue Liu,[1, 2, 3, 4] Jiao Li,[1, 2, 3, 4] Xuesong Yang,[5] Xiaojie Li,[4] Jing Kong,[4] Dongyuan Qi,[6] Fuyin Zhang,[7] Bo Sun,[7] Yuehua Liu [2, 3] *, Tingjiao Liu[1, 2] *

[1]*Department of Oral Pathology, Shanghai Stomatological Hospital & School of Stomatology, Fudan University, Tianjin Road No.2, Huangpu District, Shanghai 200001, China.*

[2]*Shanghai Key Laboratory of Craniomaxillofacial Development and Diseases, Fudan University, Tianjin Road No.2, Huangpu District, Shanghai 200001, China.*

[3]*Department of Orthodontics, Shanghai Stomatological Hospital & School of Stomatology, Fudan University, East Beijing Road No.356, Huangpu District, Shanghai 200001, China.*

[4]*School of Stomatology, Dalian Medical University, West Section No.9, South Road of Lvshun, Dalian 116044, China.*

[5]*Department of Biochemistry and Molecular Biology, Liaoning Provincial Core Lab of Glycobiology and Glycoengineering, Dalian Medical University, West Section No.9, South Road of Lvshun, Dalian 116044, China.*

[6]*Department of Oral Surgery, the First Affiliated Hospital of Dalian Medical University, No.222 Zhongshan Road, Dalian 116023, China.*

[7]*Department of Oral Surgery, the Second Affiliated Hospital of Dalian Medical University, No.467 Zhongshan Road, Dalian 116023, China.*

* Correspondence: tingjiao_liu@fudan.edu.cn; liuyuehua@fudan.edu.cn

[1]Department of Oral Pathology, Shanghai Stomatological Hospital & School of Stomatology, Fudan University, Tianjin Road No.2, Huangpu District, Shanghai 200001, China.

Supplementary figures

Figure S1

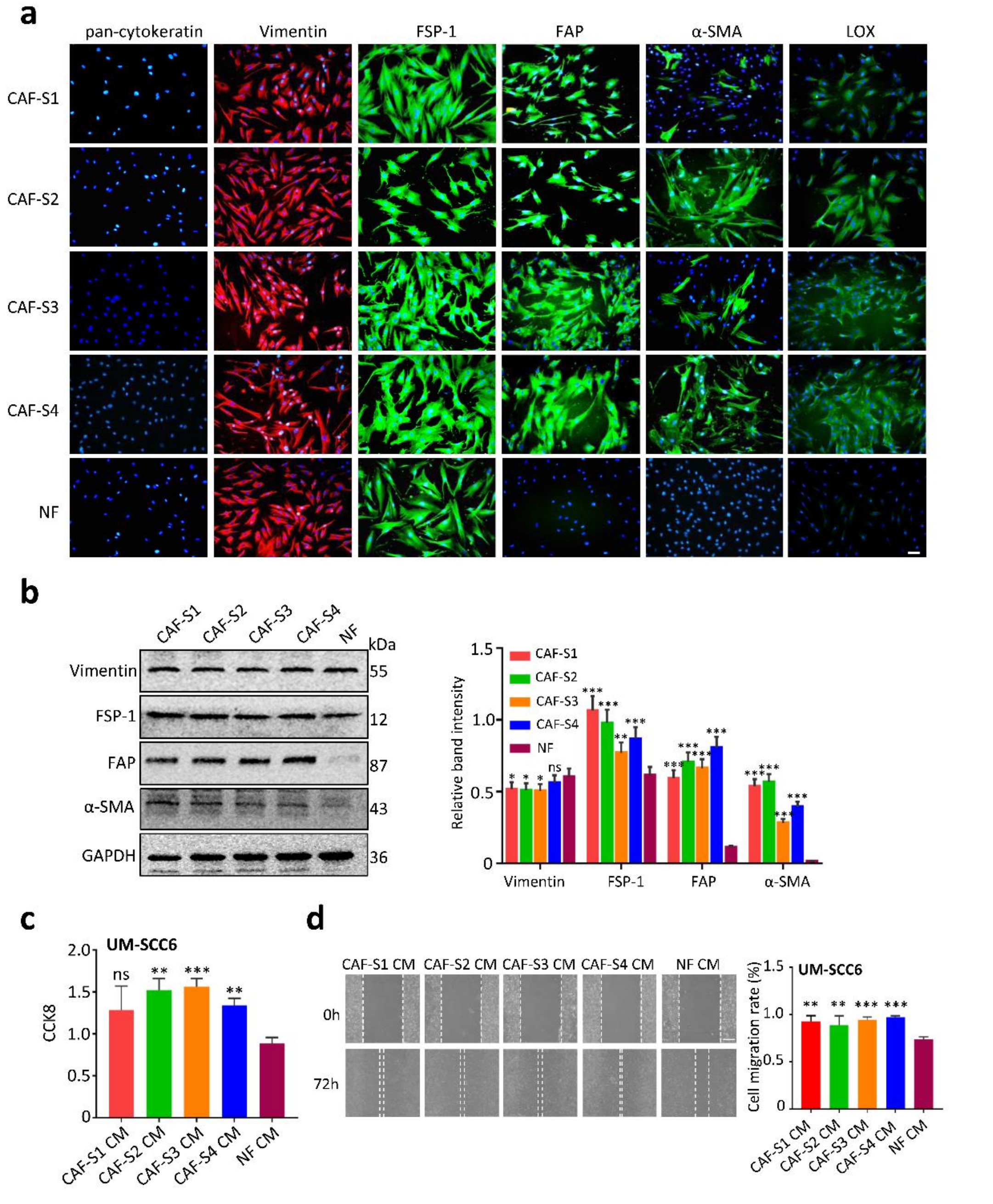

Fig. S1 The biological functions of CAFs.

(a) Immunofluorescent staining for pan-cytokeratin, vimentin, FSP-1, FAP, α-SMA and LOX in CAF-S1/S2/S3/S4 and NF. Scale bar = 50 μm. (b) Western blot analysis of vimentin, FSP-1, FAP and α-SMA in CAF-S1/S2/S3/S4 and NF. Left: images of protein bands. Right: quantification results. (c) CCK-8 assay to compare UM-SCC6 proliferation induced by CAF-S1/S2/S3/S4 and NF CM. (d) Wound healing assay to evaluate UM-SCC6 migration abilities induced by CAF-S1/S2/S3/S4 and NF CM. Scale bar = 200 μm. Left: Images of UM-SCC6 migration induced by the indicated CM. Right: quantification analysis of UM-SCC6 migration rate. For blots source data, see Fig. S11. *ns*, not significance; * $P < 0.05$; ** $P < 0.01$; *** $P < 0.001$.

Figure S2

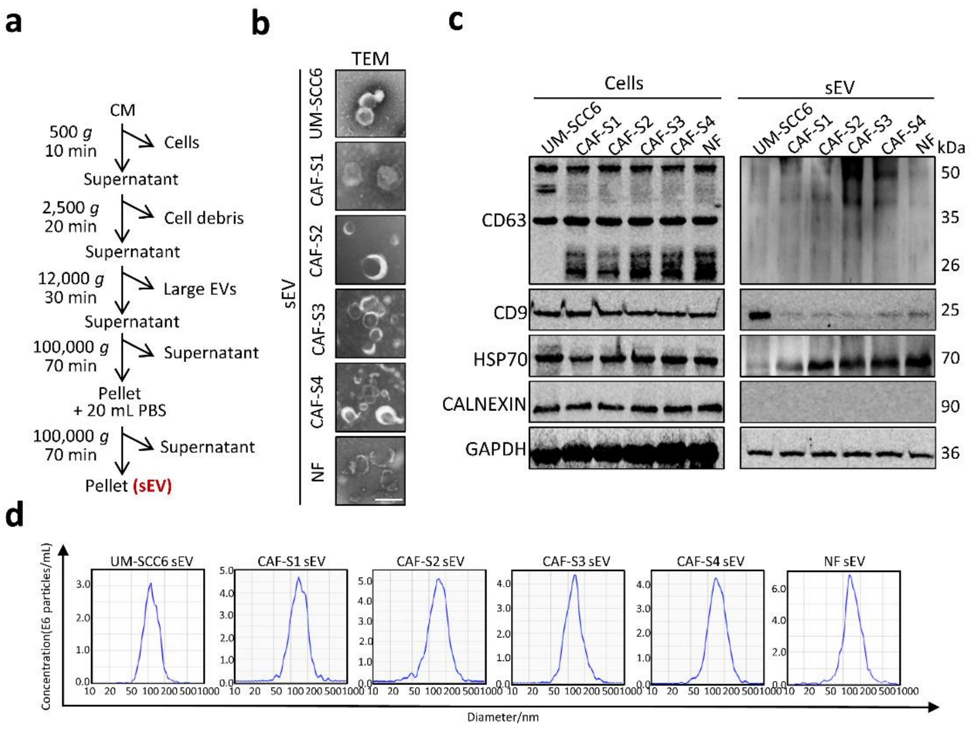

sEV	Average size (nm)	Concentration (×10^7/mL CM)
UM-SCC6	112.3	3.1
CAF-S1	136.0	5.6
CAF-S2	141.8	6.8
CAF-S3	124.5	4.8
CAF-S4	144.0	5.4
NF	142	8.0

Fig. S2 sEV separation and characteristics.

(a) Steps of sEV separation. (b) TEM images of sEVs. Scale bar = 100 nm. (c) Western blot analysis of CD63, CD9, HSP70, and CALNEXIN in sEVs. (d) Size distribution and concentration of sEVs. For blots source data, see Fig. S11.

Figure S3

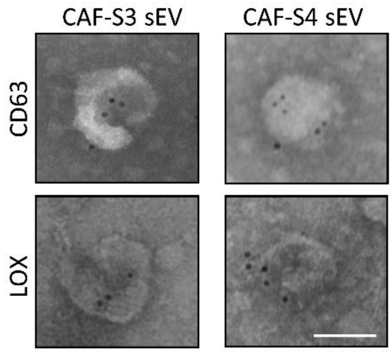

Fig. S3 LOX located on the surface of CAF-derived sEV.

Immunogold labeling of CD63 and LOX co-located on the surface of CAF-S3/S4 sEV. Scale bar = 100 nm.

Figure S4

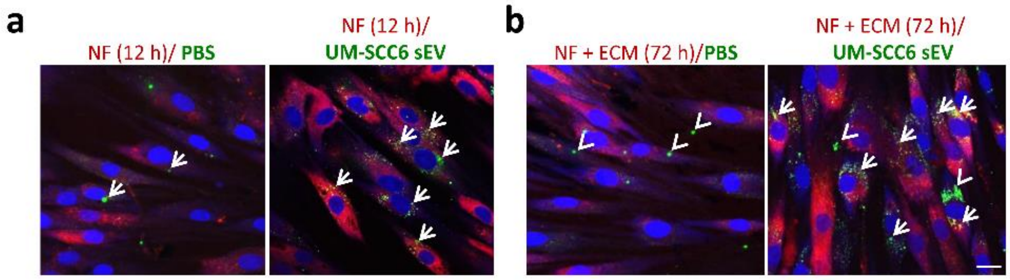

Fig. S4 Interaction of UM-SCC6 sEVs with NFs and ECM.

(a) NFs (red) were cultured for 12 h, then incubated with PBS or UM-SCC6 sEV (green) for another 12 h. sEVs were internalized into NFs (red) in large numbers (arrows). (b) NF (red) were cultured for 72 h, then incubated with PBS or UM-SCC6 sEV (green) for another 12 h. sEV were mostly internalized into NFs (arrows). Small numbers of sEVs bound to the ECM (arrowheads). Scale bar = 10 μm.

Figure S5

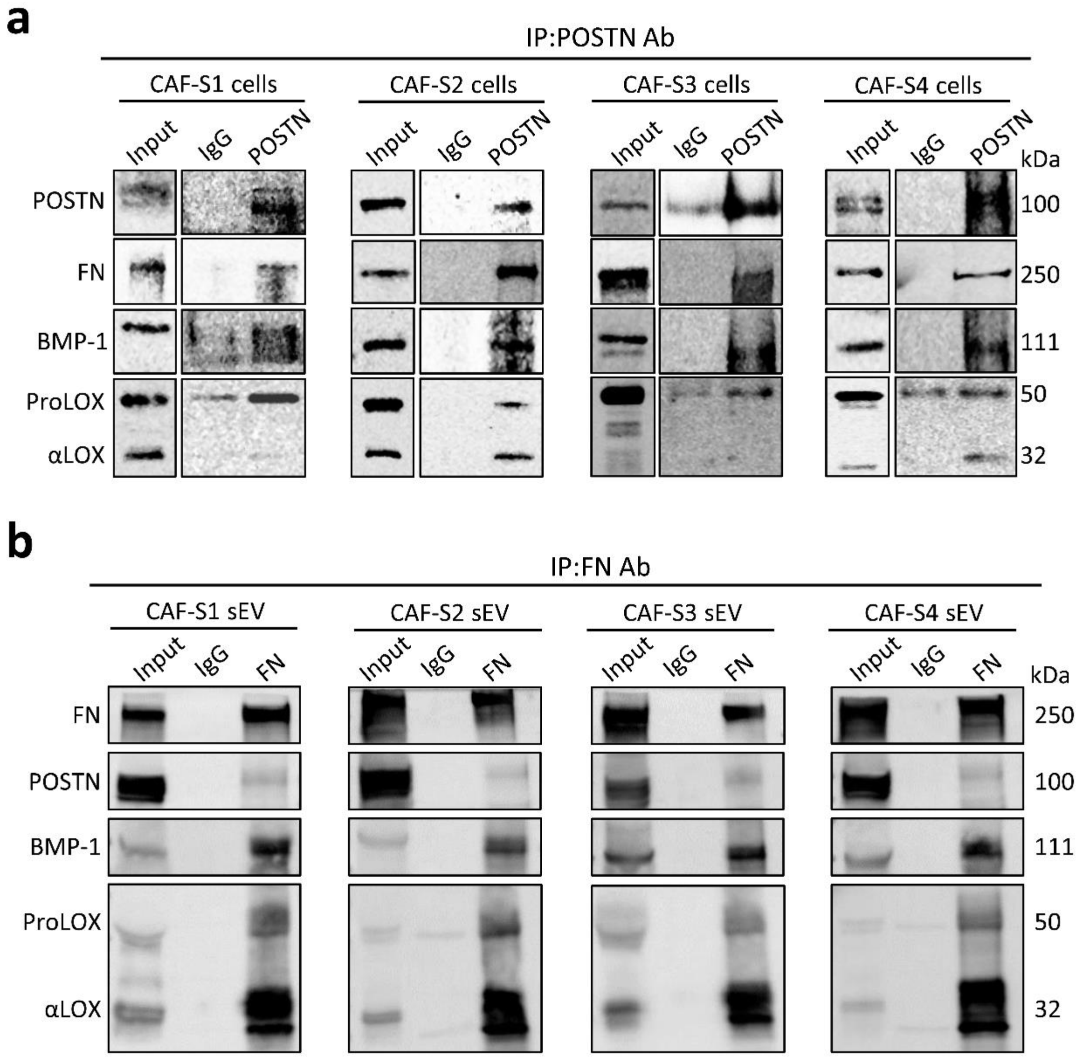

Fig. S5 POSTN/FN interaction with BMP-1 and LOX in CAFs and their sEVs.

(a, b) IP examination of POSTN/FN interaction with BMP-1 and αLOX in CAF-S1/S2/S3/S4 (a) and their sEV(b). For blots source data, see Fig. S11.

Figure S6

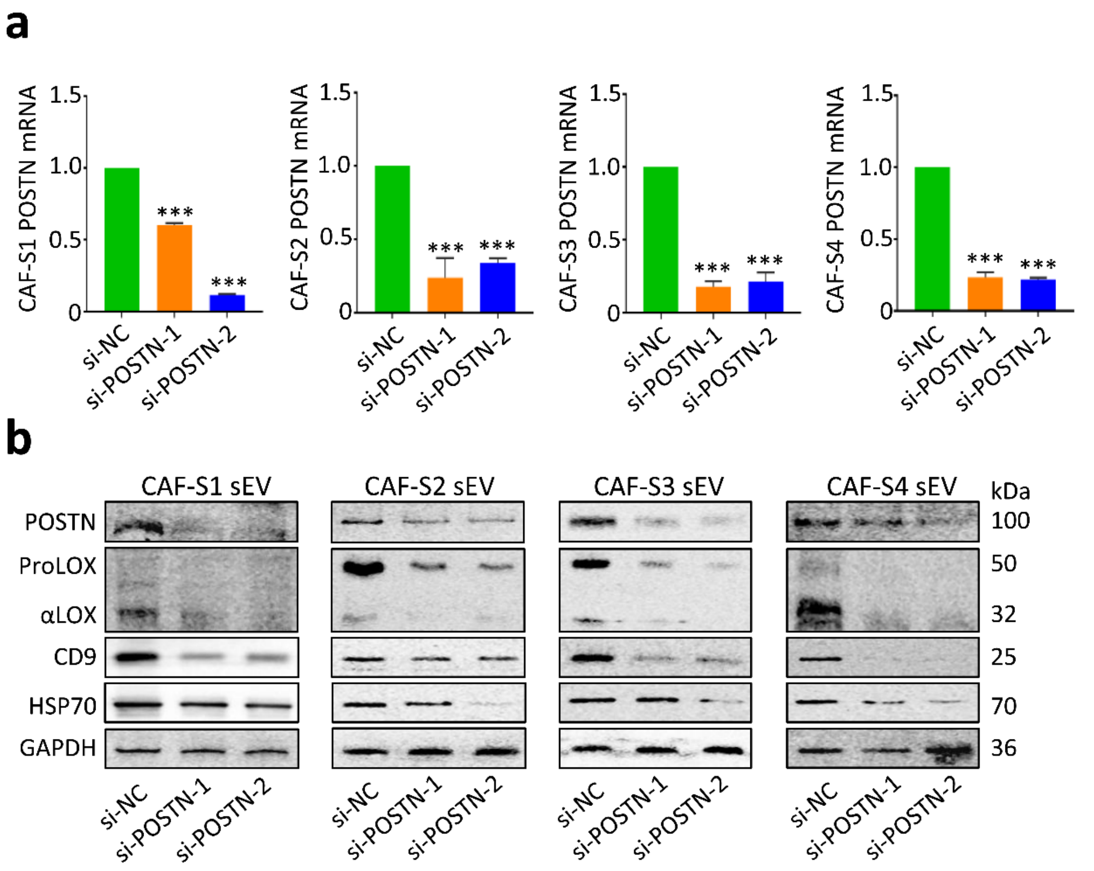

Fig. S6 Downregulation of POSTN in CAF-S1/S2/S3/S4 and their sEVs by RNA interference. (a) qRT-PCR analysis confirmed the downregulation of POSTN mRNA expression in CAF-S1/S2/S3/S4 induced by transfection with si-POSTN-1 or si-POSTN-2 compared with si-NC (n = 3 per group). (b) Western blot analysis demonstrated that transfection with si-POSTN-1 or si-POSTN-2 downregulated the expression of POSTN and αLOX in CAF-S1/S2/S3/S4 sEV compared with that in cells transfected with si-NC (n = 3 per group). CD9 and HSP70 were used as CAF sEV markers. For blots source data, see Fig. S11. ***$P < 0.001$.

Figure S7

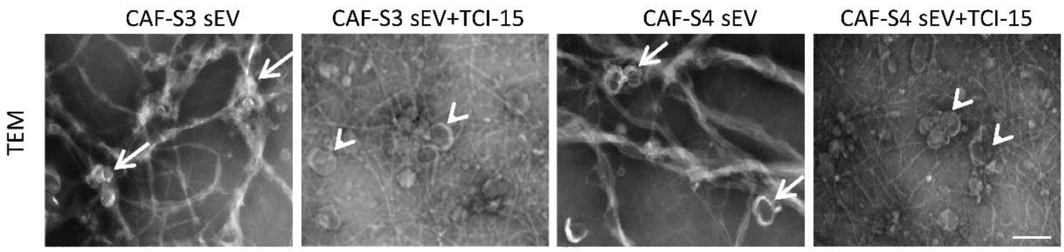

Fig. S7 TEM images of collagen crosslinking induced by CAF-S3/S4 sEVs.

Thick collagen fibers associated with sEVs (arrows) were observed. TC I-15 treatment inhibited CAF sEV-induced collagen crosslinking (arrowheads). Scale bar = 100 nm.

Figure S8

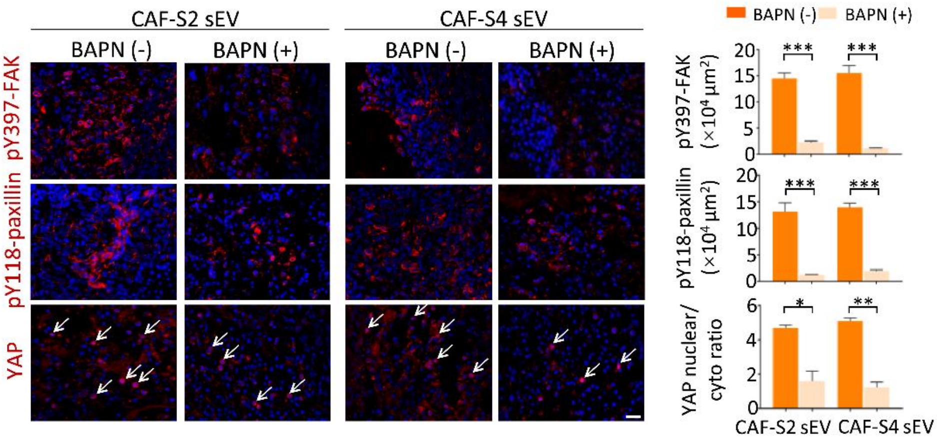

Fig. S8 Detection of FAK/paxillin/YAP pathway in UM-SCC6 xenografts treated with CAF sEVs with or without BAPN.

Expression of pY397-FAK, pY118-paxillin and YAP in UM-SCC6 xenografts (n = 4 per group). Nuclear localization of YAP (white arrows) in UM-SCC6 xenografts. Left, representative images. (Scale bar = 10 μm). Right, quantification results. *$P < 0.05$; **$P < 0.01$; ***$P < 0.001$.

Figure S9

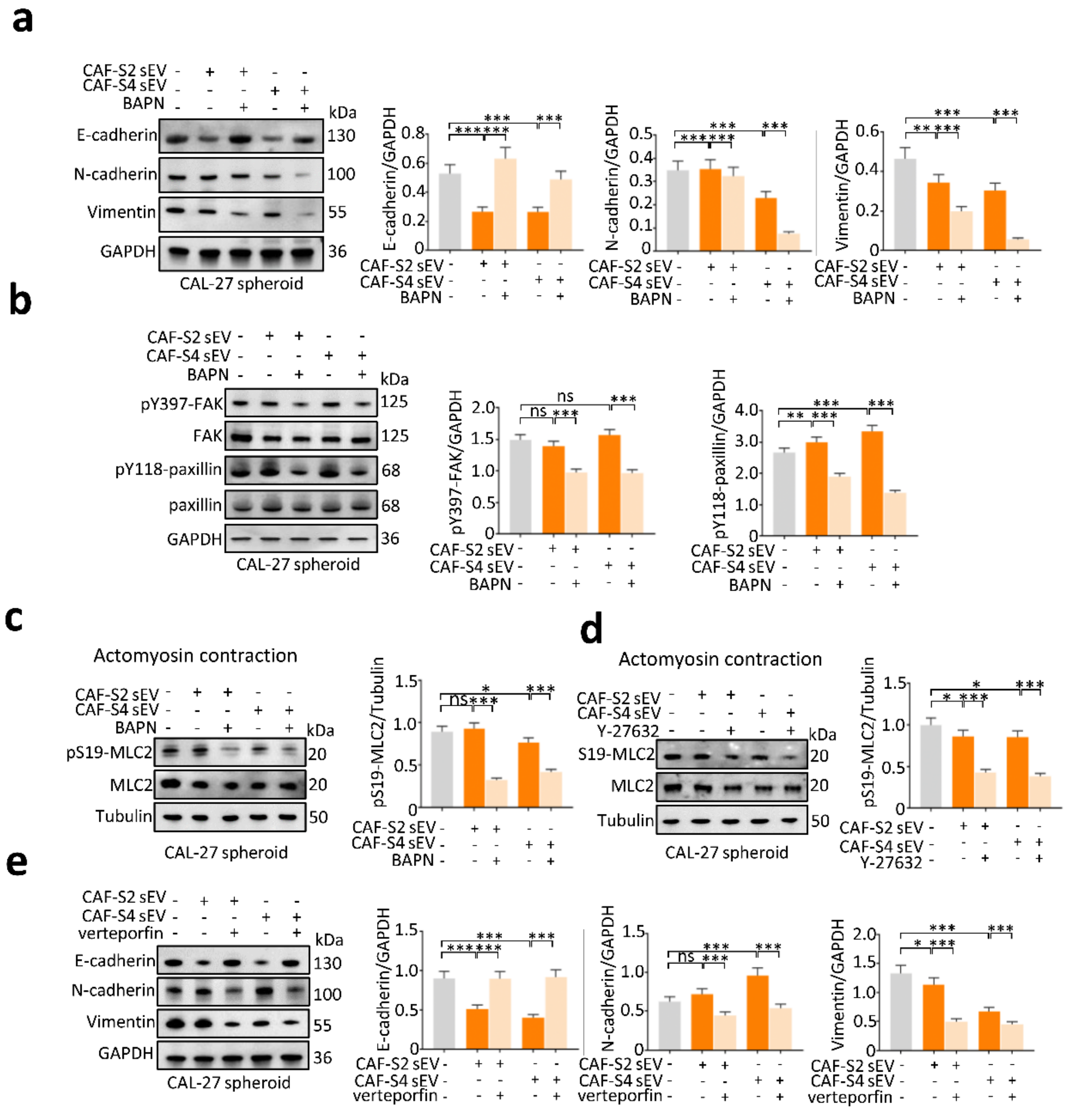

Fig. S9 EMT of CAL-27 spheroid treated with CAF sEVs activated FAK/paxillin pathway. (a) Western blot analysis of E-cadherin, N-cadherin and vimentin expression in CAL-27 spheroids treated with CAF-S2/S4 sEV with or without BAPN. (b) Expression of pY397-FAK, pY118-paxillin in CAL-27 spheroids treated with CAF-S2/S4 sEV with or without BAPN. (c) Expression of pS19-MLC2 in CAL-27 spheroids treated with CAF-S2/S4 sEV with or without BAPN. (d) Expression of pS19-MLC2 in CAL-27 spheroids treated with CAF-S2/S4 sEV with or without Y-27632. (e) Expression of E-cadherin, N-cadherin and vimentin in CAL-27 spheroids treated with CAF-S2/S4 sEV with or without verteporfin. PBS was used as a control. For blots source data, see Fig. S11. *ns,* not significance; *$P < 0.05$; **$P < 0.01$; ***$P < 0.001$.

Figure S10

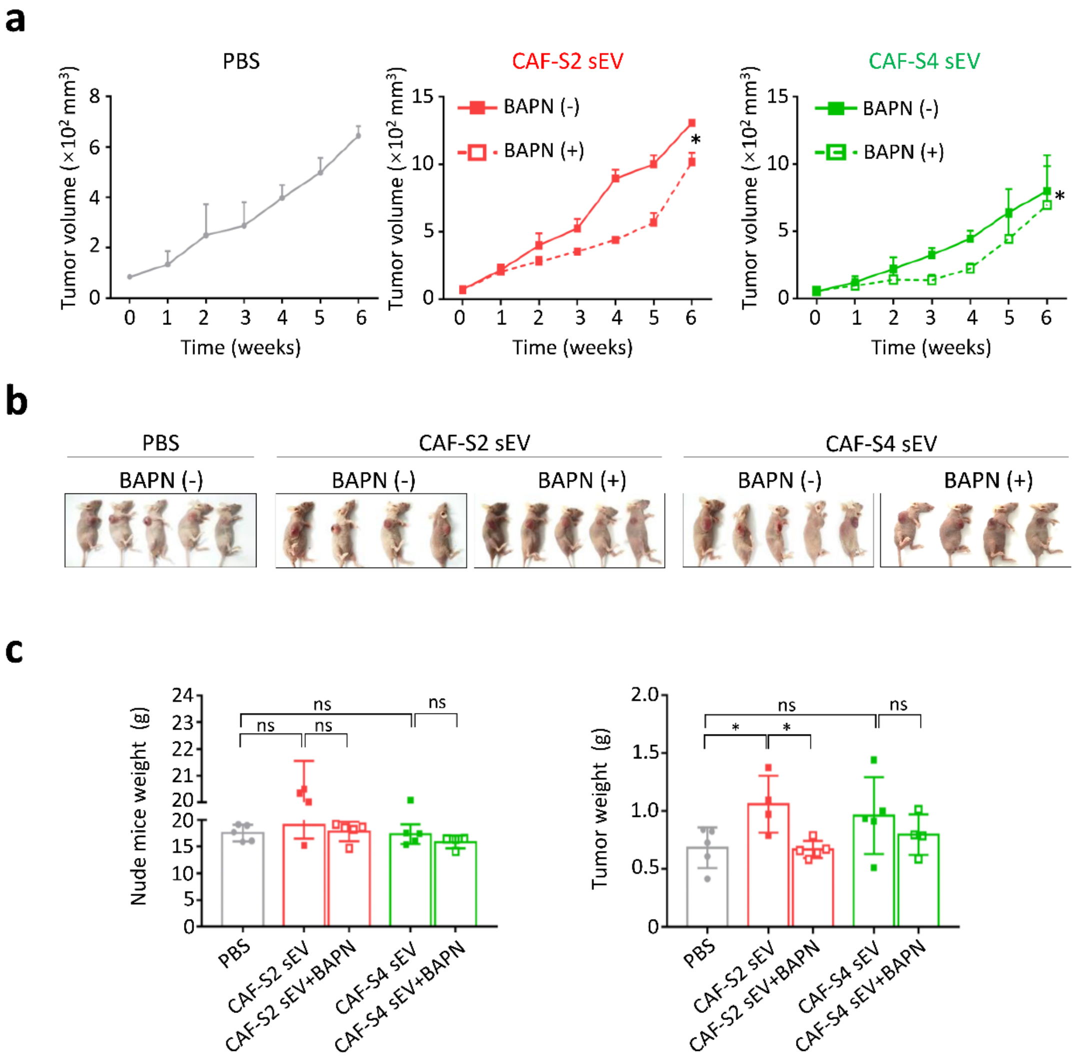

Fig. S10 Examination of UM-SCC6 xenografts treated with CAF sEVs with or without BAPN. (a) The volumes of UM-SCC6 xenografts in the CAF-S2/S4 sEV group were significantly larger than those in the CAF-S2/S4 sEVs+ BAPN group. (b) Photos of nude mice with subcutaneous xenografts in different groups. (c) Weights of nude mice and xenografts. Mouse weight did not differ significantly between the CAF-S2/S4 sEV and CAF-S2/S4 sEVs + BAPN groups. BAPN treatment decreased the weight of xenograft tumors induced by CAF-S2/S4 sEVs. *ns*, not significance; **P*< 0.05.

Figure S11

Original blots of Fig. 3b

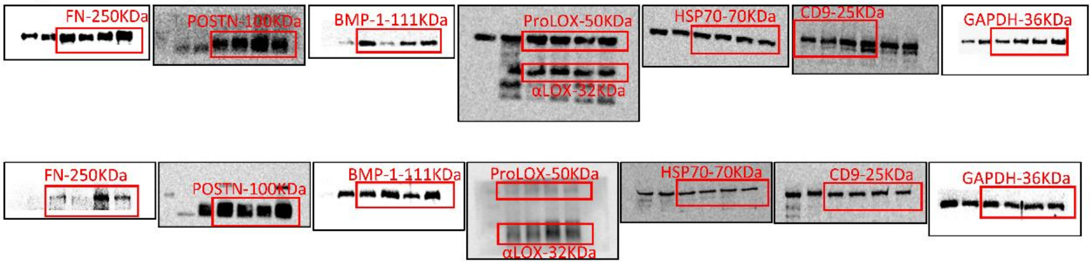

Original blots of Fig. 3c

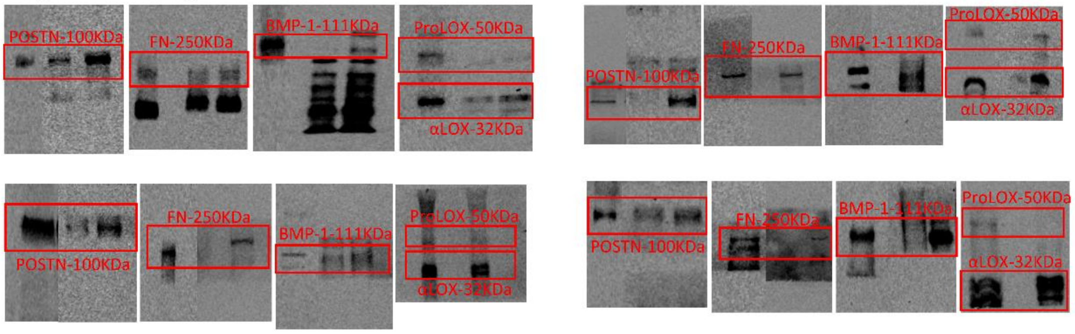

Original blots of Fig. 4b

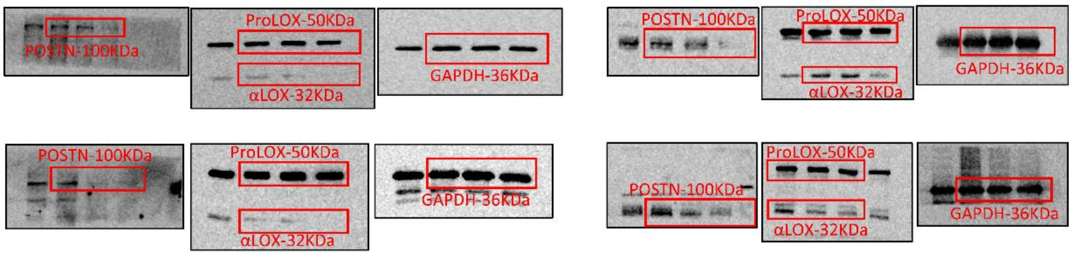

Original blots of Fig. 5b

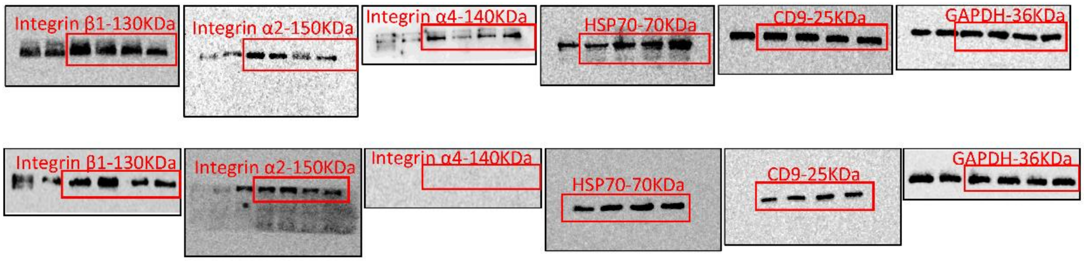

Original blots of Fig. 6c

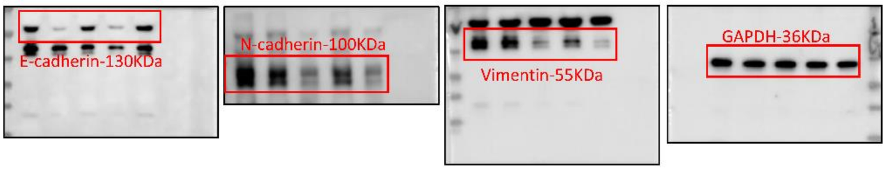

Original blots of Fig. 8b

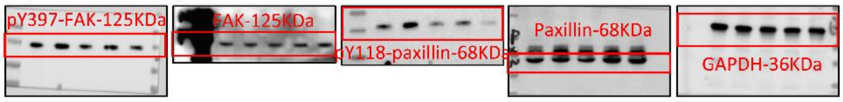

Original blots of Fig. 8c

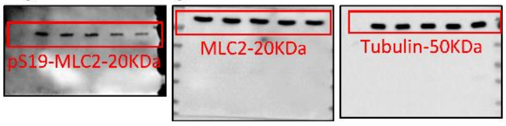

Original blots of Fig. 8d

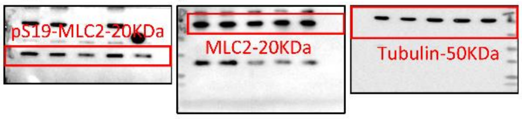

Original blots of Fig. 9b

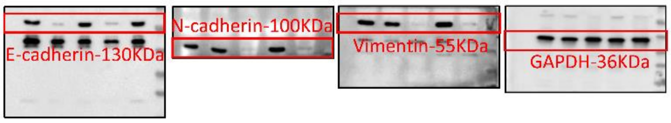

Original blots of Fig. S2b

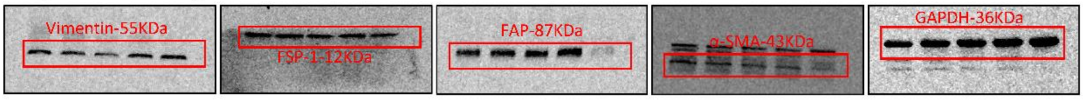

Original blots of Fig. S3c

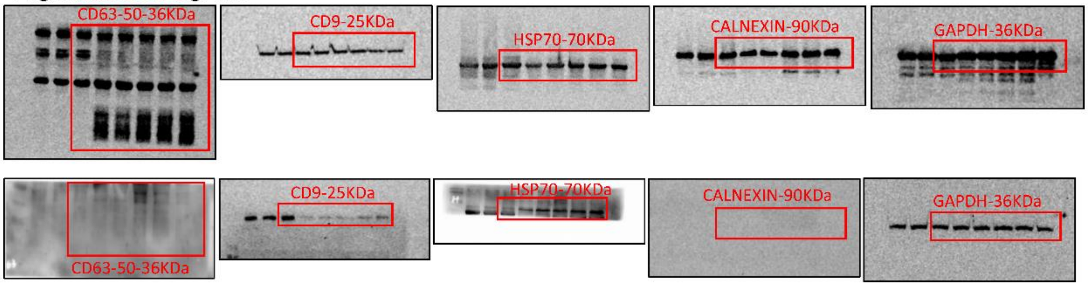

Original blots of Fig. S6a

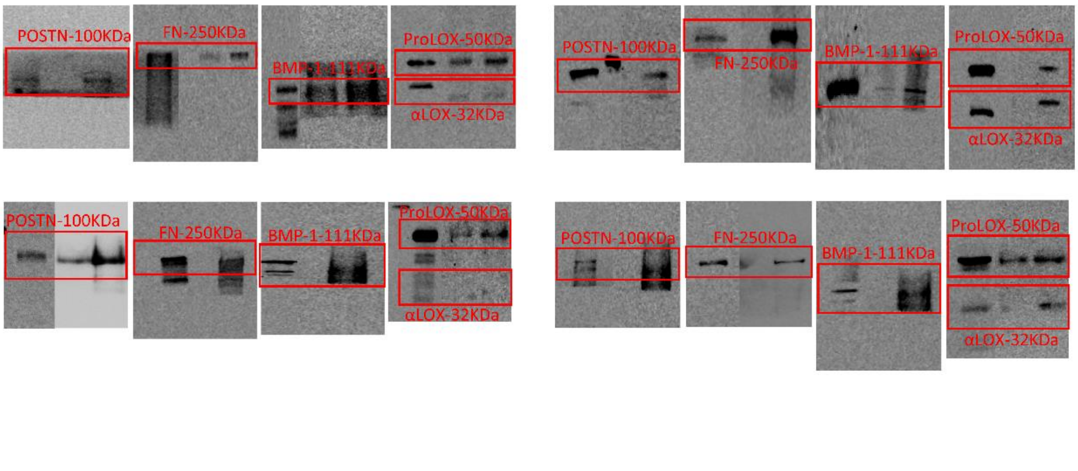

Original blots of Fig. S6b

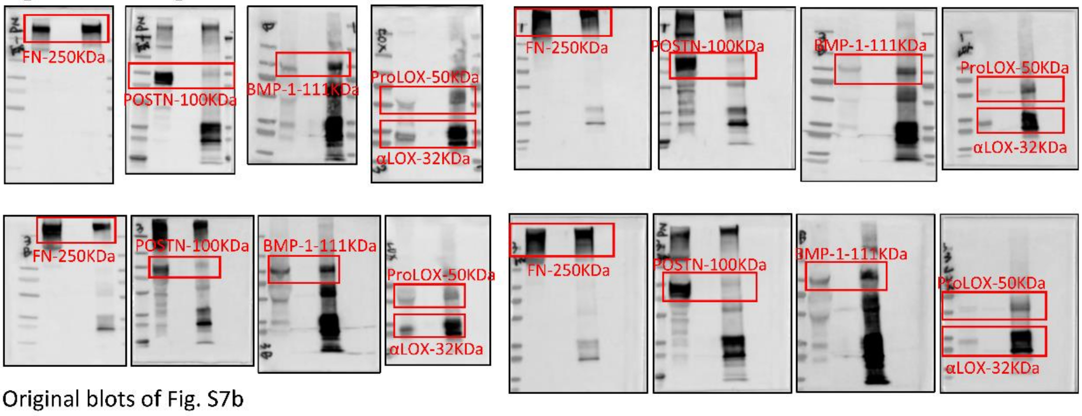

Original blots of Fig. S7b

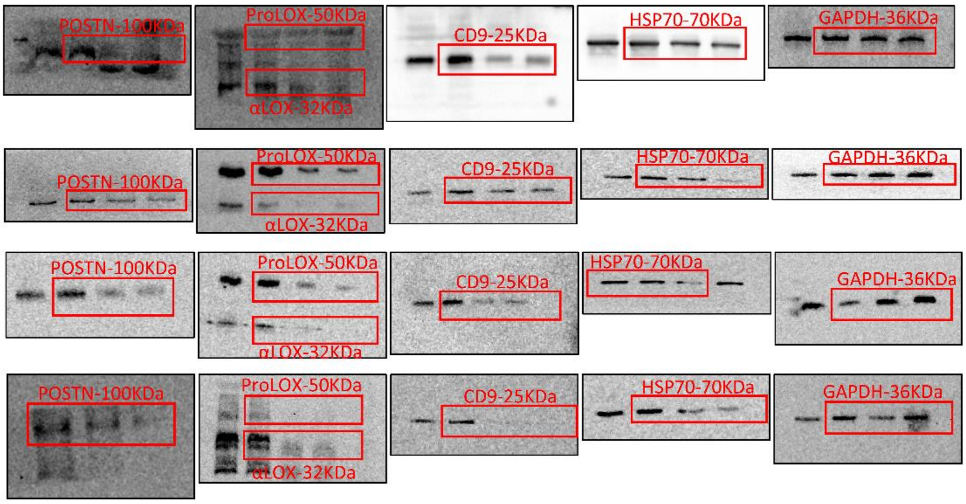

Original blots of Fig. S9a

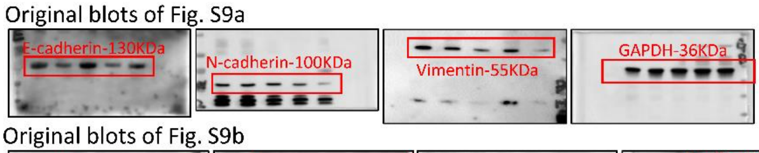

Original blots of Fig. S9b

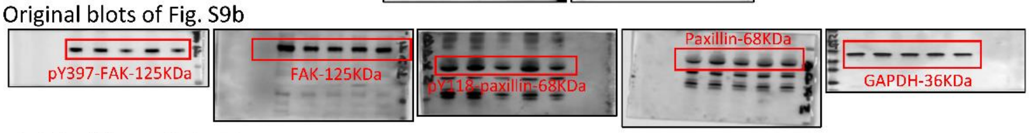

Original blots of Fig. S9c

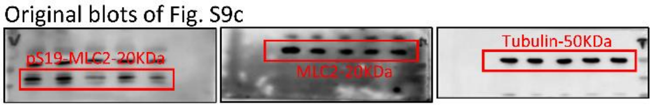

Original blots of Fig. S9d

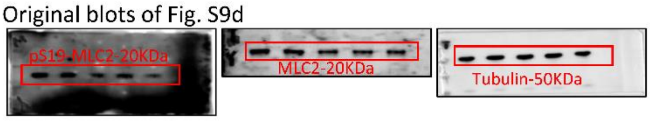

Original blots of Fig. S9e

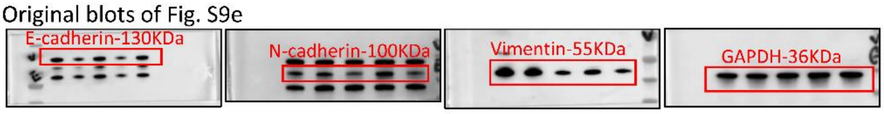

Supplement: Supplementary file 1 — Supplementary figure [file 41368_2023_236_MOESM1_ESM.pdf]
