# Supplementary material for: Carcinoma-associated fibroblast-derived lysyl oxidase-rich extracellular vesicles mediate collagen crosslinking and promote epithelial-mesenchymal transition via p-FAK/p-paxillin/YAP signaling
Source: Int J Oral Sci. 2023 Aug 2;15:32. doi: 10.1038/s41368-023-00236-1 (PMC10397209; doi:10.1038/s41368-023-00236-1)
Supplement: Supplementary file 2 — Supplementary methods [file 41368_2023_236_MOESM2_ESM.pdf]

Carcinoma-associated fibroblast-derived lysyl oxidase-rich extracellular vesicles mediate collagen crosslinking and promote epithelial-mesenchymal transition via p-FAK/p-paxillin/ YAP signaling

Xue Liu,[1, 2, 3, 4] Jiao Li,[1, 2, 3, 4] Xuesong Yang,[5] Xiaojie Li,[4] Jing Kong,[4] Dongyuan Qi,[6] Fuyin Zhang,[7] Bo Sun,[7] Yuehua Liu [2, 3] *, Tingjiao Liu[1, 2] *

[1]*Department of Oral Pathology, Shanghai Stomatological Hospital & School of Stomatology, Fudan University, Tianjin Road No.2, Huangpu District, Shanghai 200001, China.*

[2]*Shanghai Key Laboratory of Craniomaxillofacial Development and Diseases, Fudan University, Tianjin Road No.2, Huangpu District, Shanghai 200001, China.*

[3]*Department of Orthodontics, Shanghai Stomatological Hospital & School of Stomatology, Fudan University, East Beijing Road No.356, Huangpu District, Shanghai 200001, China.*

[4]*School of Stomatology, Dalian Medical University, West Section No.9, South Road of Lvshun, Dalian 116044, China.*

[5]*Department of Biochemistry and Molecular Biology, Liaoning Provincial Core Lab of Glycobiology and Glycoengineering, Dalian Medical University, West Section No.9, South Road of Lvshun, Dalian 116044, China.*

[6]*Department of Oral Surgery, the First Affiliated Hospital of Dalian Medical University, No.222 Zhongshan Road, Dalian 116023, China.*

[7]*Department of Oral Surgery, the Second Affiliated Hospital of Dalian Medical University, No.467 Zhongshan Road, Dalian 116023, China.*

* Correspondence: tingjiao_liu@fudan.edu.cn; liuyuehua@fudan.edu.cn

[1]Department of Oral Pathology, Shanghai Stomatological Hospital & School of Stomatology, Fudan University, Tianjin Road No.2, Huangpu District, Shanghai 200001, China.

Supplementary methods

CCK-8 assay

Cell viability was measured by CCK-8 assay. Cells were seeded in a 96-well plate (1000 cells/well) and cultured overnight followed by treatment with CAF or NF CM for 72 h. CCK-8 reagent (Biosharp, Shanghai, China) mixed with DMEM/F12 was added in each well and incubated for 1 h. Then, the absorbance at 450 nm was measured using a microplate reader (Bio-Rad, Hercules, CA, USA). Each well was repeated at least 3 times, and the mean absorbance was used to assess cell proliferation.

Wound healing assay

Cells were seeded into a six-well plate (5×10^5 cells per well). Next day, a wound was created in each well using a P1000 pipette tip. Then, cells were treated with CAF and NF CM for 72 h. The wound area at the start and end of each experiment was recorded using an inverted microscopy (Olympus IX71). Each well was repeated at least 3 times, and the mean migration rate was used to assess cell migration ability.

Nanoparticle Tracking analysis (NTA)

Pelleted sEVs were resuspend in PBS. The concentration and size distribution of CAF-S1/S2/S3/S4 sEVs were analyzed using ZetaView (PERTICLE METRIX, German).
