# Supplementary material for: Carcinoma-associated fibroblast-derived lysyl oxidase-rich extracellular vesicles mediate collagen crosslinking and promote epithelial-mesenchymal transition via p-FAK/p-paxillin/YAP signaling
Source: Int J Oral Sci. 2023 Aug 2;15:32. doi: 10.1038/s41368-023-00236-1 (PMC10397209; doi:10.1038/s41368-023-00236-1)
Supplement: Supplementary file 3 — Supplementary table [file 41368_2023_236_MOESM3_ESM.pdf]

Carcinoma-associated fibroblast-derived lysyl oxidase-rich extracellular vesicles mediate collagen crosslinking and promote epithelial-mesenchymal transition via p-FAK/p-paxillin/YAP signaling

Xue Liu,[1, 2, 3, 4] Jiao Li,[1, 2, 3, 4] Xuesong Yang,[5] Xiaojie Li,[4] Jing Kong,[4] Dongyuan Qi,[6] Fuyin Zhang,[7] Bo Sun,[7] Yuehua Liu [2, 3] *, Tingjiao Liu[1, 2] *

[1]*Department of Oral Pathology, Shanghai Stomatological Hospital & School of Stomatology, Fudan University, Tianjin Road No.2, Huangpu District, Shanghai 200001, China.*

[2]*Shanghai Key Laboratory of Craniomaxillofacial Development and Diseases, Fudan University, Tianjin Road No.2, Huangpu District, Shanghai 200001, China.*

[3]*Department of Orthodontics, Shanghai Stomatological Hospital & School of Stomatology, Fudan University, East Beijing Road No.356, Huangpu District, Shanghai 200001, China.*

[4]*School of Stomatology, Dalian Medical University, West Section No.9, South Road of Lvshun, Dalian 116044, China.*

[5]*Department of Biochemistry and Molecular Biology, Liaoning Provincial Core Lab of Glycobiology and Glycoengineering, Dalian Medical University, West Section No.9, South Road of Lvshun, Dalian 116044, China.*

[6]*Department of Oral Surgery, the First Affiliated Hospital of Dalian Medical University, No.222 Zhongshan Road, Dalian 116023, China.*

[7]*Department of Oral Surgery, the Second Affiliated Hospital of Dalian Medical University, No.467 Zhongshan Road, Dalian 116023, China.*

* Correspondence: tingjiao_liu@fudan.edu.cn; liuyuehua@fudan.edu.cn

[1]Department of Oral Pathology, Shanghai Stomatological Hospital & School of Stomatology, Fudan University, Tianjin Road No.2, Huangpu District, Shanghai 200001, China.

Supplementary table

Table S1a Clinical and pathological parameters in patients with OSCC

Characteristics	Cases
**Gender**	
Male	16
Female	14
**Age**	
$\leq 62\ year$	17
$\geq 62\ year$	13
**Sites**	
Palate	4
Buccal mucosa	3
tongue	8
Lip	4
gingiva	5
Mouth floor	1
Pharynx	1
Submandibular	2
Mandible	2
**Grade**	
I	14
II	11
III	5

Table S1b Correlation coefficients between LOX and clinical parameters in OSCC patients

Variables	LOX	Gender	Age	Sites	Grade
LOX	1				
Gender	-0.077	1			
Age	0.189	0.101	1		
Sites	0.211	0.255	0.037	1	
Grade	-0.171	-0.139	-0.298	-0.224	1

Note: Pearson correlation analysis was used for continuous variables; Spearman correlation analysis was used for category variables. P-value < 0.05
